# Core–Sheath Fibers via Single-Nozzle Spinneret Electrospinning of Emulsions and Homogeneous Blend Solutions

**DOI:** 10.3390/ma17215379

**Published:** 2024-11-04

**Authors:** Selin Kyuchyuk, Dilyana Paneva, Nevena Manolova, Iliya Rashkov

**Affiliations:** Laboratory of Bioactive Polymers, Institute of Polymers, Bulgarian Academy of Sciences, Acad. G. Bonchev St, Bl. 103A, BG-1113 Sofia, Bulgaria; selin.erdinch@polymer.bas.bg (S.K.); manolova@polymer.bas.bg (N.M.)

**Keywords:** emulsion electrospinning, blend solution electrospinning, self-organization, core–sheath fibers, core–double sheath fibers, core–shell fibers, antimicrobial and anticancer activity, biomedical applications, agropharmaceuticals

## Abstract

The preparation of core–sheath fibers by electrospinning is a topic of significant interest for producing composite fibers with distinct core and sheath functionalities. Moreover, in core–sheath fibers, low-molecular-weight substances or nanosized inorganic additives can be deposited in a targeted manner within the core or the sheath. Commonly, for obtaining a core–sheath structure, coaxial electrospinning is used. It requires a coaxial spinneret and suitable immiscible solvents for the inner and outer solutions. The single-nozzle spinneret electrospinning of emulsions can address these issues, but use of a stabilizing agent is needed. A third approach—preparation of core–sheath fibers by single-nozzle spinneret electrospinning of homogeneous blend solutions of two polymers or of a polymer/low-molecular-weight substance—has been much less studied. It circumvents the difficulties associated with the coaxial and the emulsion electrospinning and is thoroughly discussed in this review. The formation of core–sheath fibers in this case is attributed to phase-separation-driven self-organization during the electrospinning process. Some possibilities for obtaining core–double sheath fibers using the same method are also indicated. The gained knowledge on potential applications of core–sheath fibers prepared by single-nozzle spinneret electrospinning of emulsions and homogeneous blend solutions is also discussed.

## 1. Introduction

Electrospinning is a versatile electrohydrodynamic process that enables the preparation of continuous micro- and nanofibers by applying an electric field to a polymer solution or melt [1,2,3,4,5]. The preparation of continuous defect-free fibers is contingent upon the polymer exhibiting a molar mass and concentration that exceeds a certain threshold. This ensures that the requisite condition for fiber formation is met, namely, effective entanglement of the polymer chains in the spinning solution or melt [6]. In instances where the aforementioned condition is not met, namely when the molar mass or polymer concentration is below the prescribed limit values, no fibers are formed. However, micro- or nanoparticles are produced instead. This electrohydrodynamic process is referred to as electrospraying [1,7]. Research conducted on the applicability of electrospun non-woven textiles has demonstrated that these materials have the potential for use in a number of fields, including biomedicine, filtration and separation, the design of protective clothing and sensors, in agriculture, food packaging and preservation, and nanoelectronics [8,9,10,11,12,13,14,15,16,17,18,19,20,21,22,23,24,25,26,27,28,29,30].

From an equipment viewpoint, the first and most straightforward electrospinning technique is that which uses a single-nozzle spinneret and a static collector [31]. The use of this equipment results in preparation of random monolithic polymer fibers. The substitution of a high-speed rotating drum collector for the conventional static collector facilitates the preparation of aligned monolithic polymer fibers. Typical electrospinning equipment comprises a high-voltage source, a pump for maintaining the solution flow rate, and a syringe equipped with a single-nozzle spinneret and a rotating drum collector. A number of review articles address the conventional electrospinning equipment, as well as the parameters that affect the electrospinning process and the morphology of the monolithic fibers obtained [2,4,31,32,33]. Solution electrospinning is a more widely used than melt electrospinning. Additionally, it is the sole method for preparation of micro- or nanofibers from thermally unstable polymers. Moreover, this electrospinning technique has successfully transitioned from laboratory-scale applications to industrial-scale production. The parameters that affect the conventional electrospinning process and, consequently, the morphology of the resulting fibers can be classified into the following categories: polymer-related parameters (polymer molar mass and dispersity; polymer architecture, whether linear or branched); solution-related parameters (polymer concentration, viscosity, surface tension, conductivity); parameters of the electrospinning process [applied voltage, solution delivery rate, tip-to-collector distance, collector design, velocity rate of the rotating drum collector, spinneret design (single- or multi-nozzle one)]; and parameters of the environment (temperature, humidity, air velocity in the chamber) [4,34,35,36].

Since 2000, intensive scientific research has facilitated the accelerated development of the electrospinning method. The efforts are focused on: (i) enhancement of the productivity of the electrospinning process (fiber yield and production rate); (ii) preparation of non-woven textile with desired complex architecture; and (iii) preparation of fibers with a given complex architecture.

**Figure 1:**
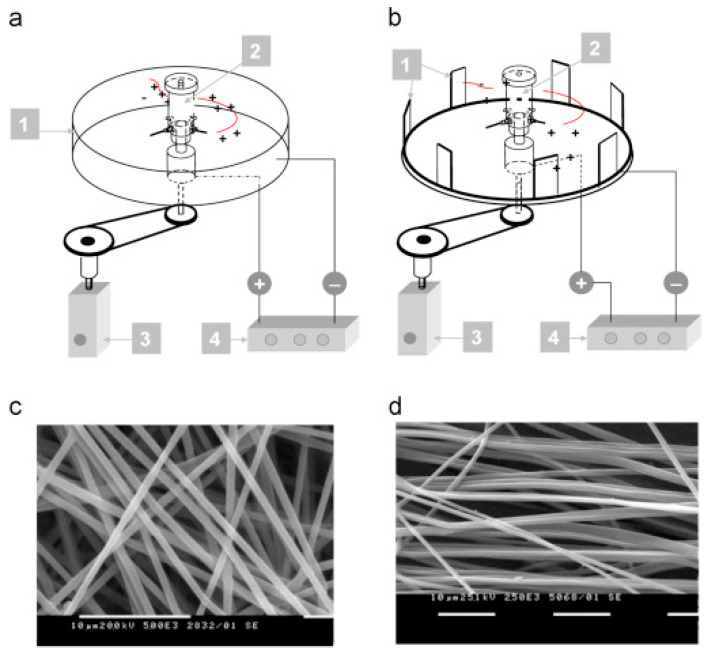
Schematic of the electrospinning setup: Apparatuses with stationary collectors (1) and a rotating feed unit (2), electric motor (3), and a high-voltage power supply (4); cylindrical collector (**a**) and collector composed of strips (**b**). SEM micrographs of mats deposited on the cylindrical collector (**c**) and obtained between the strips (**d**). Reprinted from ref. [39]. Copyright (2014), with permission from Elsevier.

With respect to the enhancement of the productivity of the electrospinning process, a number of adaptations to the electrospinning equipment have been developed that enable multi-jet electrospinning [31,37]. The latter is divided into two main types: multi-nozzle spinneret and needleless electrospinning [31,37]. In multi-nozzle spinneret electrospinning, a number of feeding solutions are fed simultaneously through a series of single-nozzle spinnerets, and the resulting fibers are subsequently deposited on a common collector [31]. The development of the needleless electrospinning has led to a substantial rise in the manufacture of non-woven fabrics. It comprises the electrospinning of a polymer directly from an open liquid surface [37]. The needleless electrospinning technique has been adopted by various industries [38]. The technology is currently being commercialized by Elmarco s.r.o. (Liberec, Czech Republic) under the brand name “Nanospider”. The combination of multi-nozzle spinneret electrospinning and centrifugal field generation has also been identified as an effective tool for enhancement of the productivity of the electrospinning process and reducing the time required for the preparation of non-woven textiles via electrospinning [31,39,40]. A purposely designed multi-nozzle spinneret device rotates at high velocity, and the cylindrical collector remains stationary (Figure 1a). This equipment configuration enables the production of a non-woven textile composed of random monolithic polymer fibers (Figure 1c). The purposely designed stationary collector consisting of circularly arranged metal strips (Figure 1b) has enabled the efficient preparation of a non-woven textile composed of aligned fibers (Figure 1d).

**Scheme 1:**
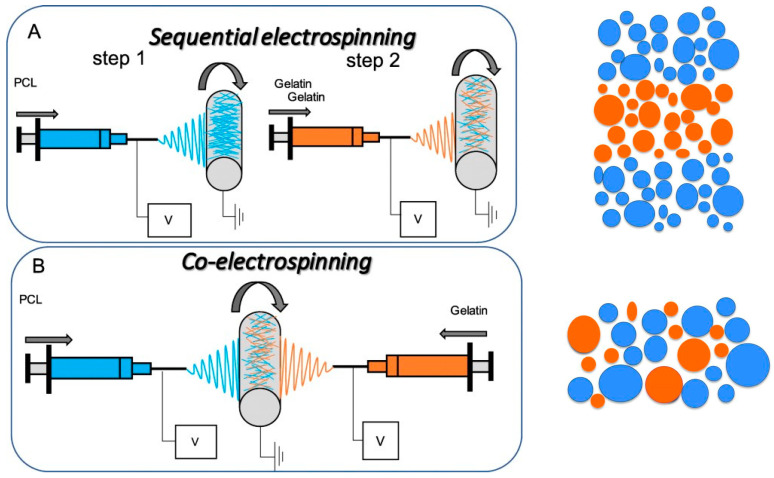
Schematic of sequential and co-electrospinning setup where the position of the needles can be next or opposite to each other: (**A**) Sequential setup can be used to fabricate layered; (**B**) co-electrospinning setup can be used to fabricate mixed fibrous structure [34]. Cross sections of the resulting non-woven prepared by both techniques are also shown. PCL—poly(ε-caprolactone). Adapted with permission from ref. [43]. Copyright 2021, Science Partner Journals (SPJ).

The preparation of a composite non-woven textile with complex architecture from different polymers enables the combination and/or improvement of the properties of the individual polymers. Two electrospinning techniques have been developed for the preparation of a composite non-woven textile with complex architecture: sequential electrospinning and simultaneous electrospinning. Double- or multi-layered non-woven textiles are prepared by sequential electrospinning or one-by-one electrospinning. It consists of electrospinning of a spinning solution of a polymer which forms a layer with a specific functionality, followed by electrospinning of a solution of another polymer which forms a second layer with a distinct functionality (Scheme 1A). This method uses conventional electrospinning equipment. It is possible to prepare double-layered fibrous materials, as well as sandwich-like fibrous materials with alternating layers (Scheme 1A). In the case of simultaneous electrospinning (Scheme 1B), two different single-nozzle spinnerets are used, through which two spinning solutions of two different polymers are fed simultaneously, subjected to high voltage, and the resulting fibers are deposited on a common collector [41,42]. The resulting non-woven textile is composed of interwoven micro- or nanofibers derived from the different polymers. A unifying term for this electrospinning technique is still lacking. Simultaneous electrospinning is also referred to as co-electrospinning, or dual electrospinning. Noteworthy, the term co-electrospinning has also been used to describe blend, emulsion, or coaxial electrospinning. In our opinion, the terms simultaneous electrospinning and dual electrospinning are much more appropriate to distinguish this electrospinning technique from blend, emulsion, or coaxial electrospinning. The potential of simultaneous electrospinning for the preparation of fibers loaded with two drugs, which may interact chemically if they are in a common solution, has been demonstrated [41]. The feasibility of fabricating mats with enhanced mechanical properties has been demonstrated through the simultaneous electrospinning of two solutions comprising the high-melting polylactide (PLA) and the low-melting poly(ε-caprolactone) (PCL), respectively, followed by annealing of the resulting composite fibrous materials [42].

**Scheme 2:**
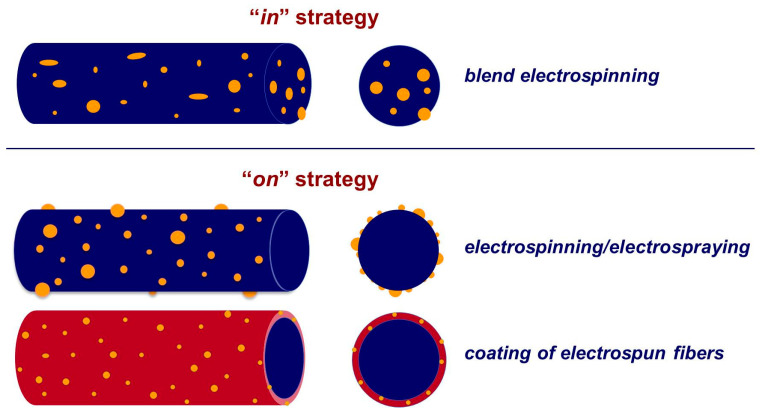
Schematic representation of a longitudinal and transverse section of a fiber obtained by applying the “*in*” and “*on*” strategies and the corresponding techniques for preparation of fibers with different architectures based on polymer/polymer, polymer/oligomer, or polymer/bioactive substance pairs. The additive of a second polymer, oligomer, or low-molecular-weight bioactive substance is indicated by yellow circles. For the coating technique, the polymer coating of an electrospun fiber is illustrated in red.

The possibility of combining pairs, including polymer/polymer, polymer/oligomer, and polymer/low-molecular-weight substances, which exhibit distinct natures and properties, at the level of a single micro- or nanofiber through the electrospinning process is of particular interest. In order to prepare these architecturally complex fibers, two strategies, designated here for brevity as the “*in*” and “*on*” strategies, are applied (Scheme 2). The “*in*” strategy uses blend electrospinning technique, whereby a solution comprising a fiber-forming polymer and an additive of a second, distinct polymer, oligomer, or bioactive substance is simultaneously processed. The application of this strategy results in the preparation of fibers comprising a continuous phase of the fiber-forming polymer and a dispersed phase of the second polymer, oligomer, or low-molecular-weight bioactive substance. This additive is predominantly situated within the fiber bulk. In order to prepare fibers in which the aforementioned additive is located only on the fiber surface, the “*on*” strategy is employed. The developed techniques to implement the “*on*” strategy are as follows: simultaneous electrospinning/electrospraying [41,44] and a two-stage technique consisting in coating of electrospun fibers [45].

By applying the technique of coating electrospun fibers, it is possible to prepare fibers with a core–sheath(s) structure [46]. Core–sheath(s) fibers are of particular interest for biomedical, cosmetic, and food packaging applications, as their main advantage is that the core and the sheath(s) can be selectively and separately loaded with bioactive substances [32,47,48]. Therefore, the complex architecture of this type of composite fiber can provide an excellent platform for simultaneous or sequential release of bioactive agents that are relevant to specific applications. The main part of the research on the preparation of core–sheath(s) fibers has been carried out by the coaxial electrospinning technique [2,49,50,51,52,53]. Another widely applied technique for preparation of core–sheath(s) fibers is the emulsion electrospinning [50,54,55]. It has been demonstrated that for certain pairs of polymer/polymer or polymer/low-molecular-weight substance, the single-nozzle spinneret electrospinning of their homogeneous blend solutions enables the preparation of fibers having core–sheath(s) architecture [56,57,58,59,60,61,62,63], and research in this area is scarce. Furthermore, in contrast to the numerous review articles on the coaxial and emulsion electrospinning techniques as a tool for preparation of core–sheath fibers, no review article exists that provides a comprehensive summary of the advancements made in the design of core–sheath(s) fibers by single-nozzle spinneret electrospinning of homogeneous blend solutions.

The objective of this review is to summarize the gained knowledge on the preparation of core–sheath(s) fibers by single-nozzle spinneret electrospinning of homogeneous blend solutions. The paper commences with a brief overview of the merits and shortcomings associated with the most commonly applied techniques for obtaining core–sheath fibers: the coaxial and emulsion electrospinning. The results on the potential applications of core–sheath(s) fibers prepared by single-nozzle spinneret electrospinning of emulsions and homogeneous blend solutions are also summarized.

## 2. Approaches for Preparation of Core–Sheath(s) by Electrospinning

### 2.1. Electrospinning Using Multi-Nozzle Spinneret (Preparation of Fibers with Core–Sheath, Core–Double Sheath or Double Core–Sheath Architecture)

The preparation of core–sheath fibers by electrospinning using a double-nozzle spinneret (commonly referred to as “coaxial electrospinning”) represents the most extensively studied approach for the fabrication of fibers exhibiting this particular architecture. In recent years, the number of review articles devoted to coaxial electrospinning has been considerable [4,46,50,64,65,66].

**Scheme 3:**
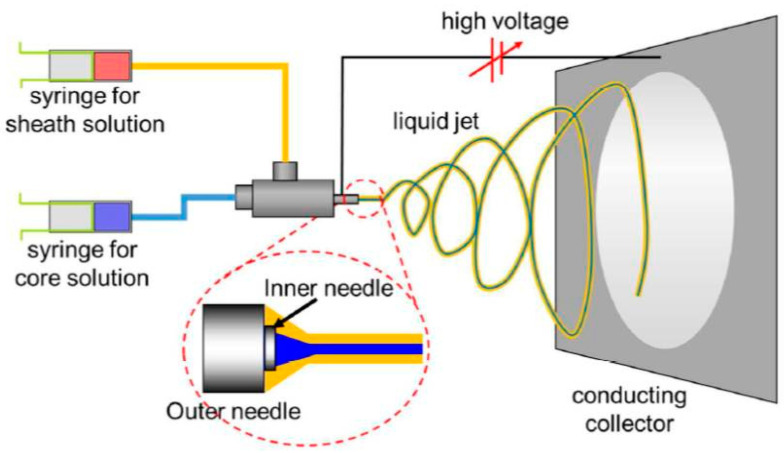
Diagram of coaxial electrospinning and resulting core–sheath fibers. Reprinted with permission from ref. [67]. Copyright (2017), American Chemical Society.

In order to conduct coaxial electrospinning, it is necessary to complicate the electrospinning equipment by including an auxiliary device that ensures the simultaneous supply of the solutions that form the core and sheath, respectively (Scheme 3). The auxiliary coaxial device is comprised of two distinct compartments, which are fed in a controlled manner with spinning solutions via two pumps. The device also features a spinneret, which is composed of two coaxially aligned nozzles. The inner nozzle is supplied with the solution that constitutes the fiber core, whereas the outer nozzle receives the solution that forms the fiber sheath. The application of a high voltage to the spinneret results in the formation of a Taylor cone, in which the polymer solution that forms the fiber sheath envelops the solution that forms the fiber core. Following the evaporation of the solvent during the electrospinning process, dry composite core–sheath fibers are formed.

An advantage of the coaxial electrospinning is that it enables the preparation of composite fibers with the participation of polymers that are not electrospinnable on their own [4,65]. Furthermore, coaxial electrospinning represents a promising technique for the fabrication of core–sheath fibers, wherein an environmentally sensitive bioactive agent is integrated into the core [64]. The sheath serves to safeguard the incorporated substance from any unintended modifications to its chemical composition or functional activity. Regarding controlled drug delivery, it is claimed that the coaxial electrospinning is an effective technique for design of new dosage forms with a tailored drug release profile. The feasibility of preparing hollow fibers via a coaxial electrospinning process, followed by the removal of the material comprising the fiber core, has also been demonstrated [63,68,69,70].

The major drawback of coaxial electrospinning is the complexity of the equipment required, necessitating the use of an auxiliary coaxial device and two pumps to supply the two spinning solutions. This complicated electrospinning equipment is a prerequisite for the increase in the parameters affecting the process and the preparation of fibers with continuous core surrounded by a continuous sheath, respectively [65]. An example of this is the core–shell flow rate ratio which is a crucial parameter for the successful preparation of uniform core–sheath fibers. Other parameters are the polymer concentration, viscosity, compatibility, and conductivity of the spinning solutions. It has been demonstrated that when electrospinnable and non-electrospinnable polymers are used, the electrospinnable polymer must be present in a sufficiently high concentration in its spinning solution to ensure the formation of uniform core–sheath fibers. Therefore, in contrast to single-nozzle spinneret electrospinning, in coaxial electrospinning, the number of parameters altering the formation of a stable composite Taylor cone, and, consequently, of uniform core–sheath fibers, increases. In order to find the optimal parameters, an empirical approach is employed.

**Figure 2:**
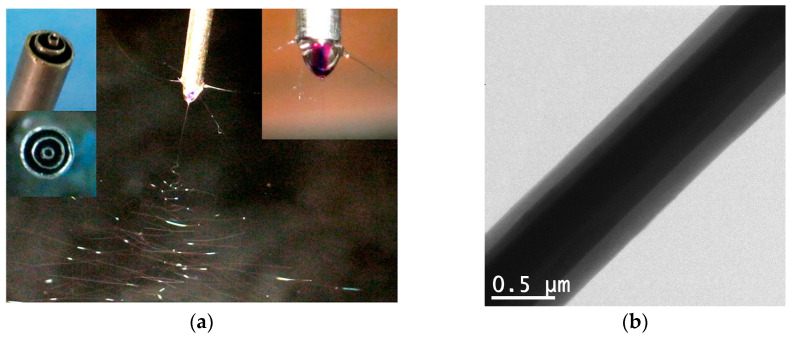
(**a**) Digital photographs of the triaxial electrospinning process. Upper-left insets show the structure of the homemade spinneret; upper-right inset shows a typical compound Taylor cone. (**b**) TEM image of a trilayer nanofiber. Reprinted with permission from ref. [71]. Copyright (2015), American Chemical Society.

Spinnerets with a design that includes three nozzles have also been developed, resulting in the production of core–double sheath fibers (triaxial electrospinning) or double core–sheath fibers. Core–double sheath fibers have been prepared using three-nozzle concentric spinneret and three distinct solutions fed by three pumps to the three-nozzle concentric spinneret (Figure 2) [71].

The key parameter that affects the preparation of uniform core–double sheath fibers by triaxial electrospinning is to provide conditions for the formation of a composite Taylor cone composed of inner, intermediate and outer laminar flows, which remain in concentric alignment throughout the electrospinning process. As noted by [4], all process parameters that are key to the formation of a stable Taylor cone in coaxial electrospinning are also valid in triaxial electrospinning, but in triaxial electrospinning their influence and control is much more complicated.

**Figure 3:**
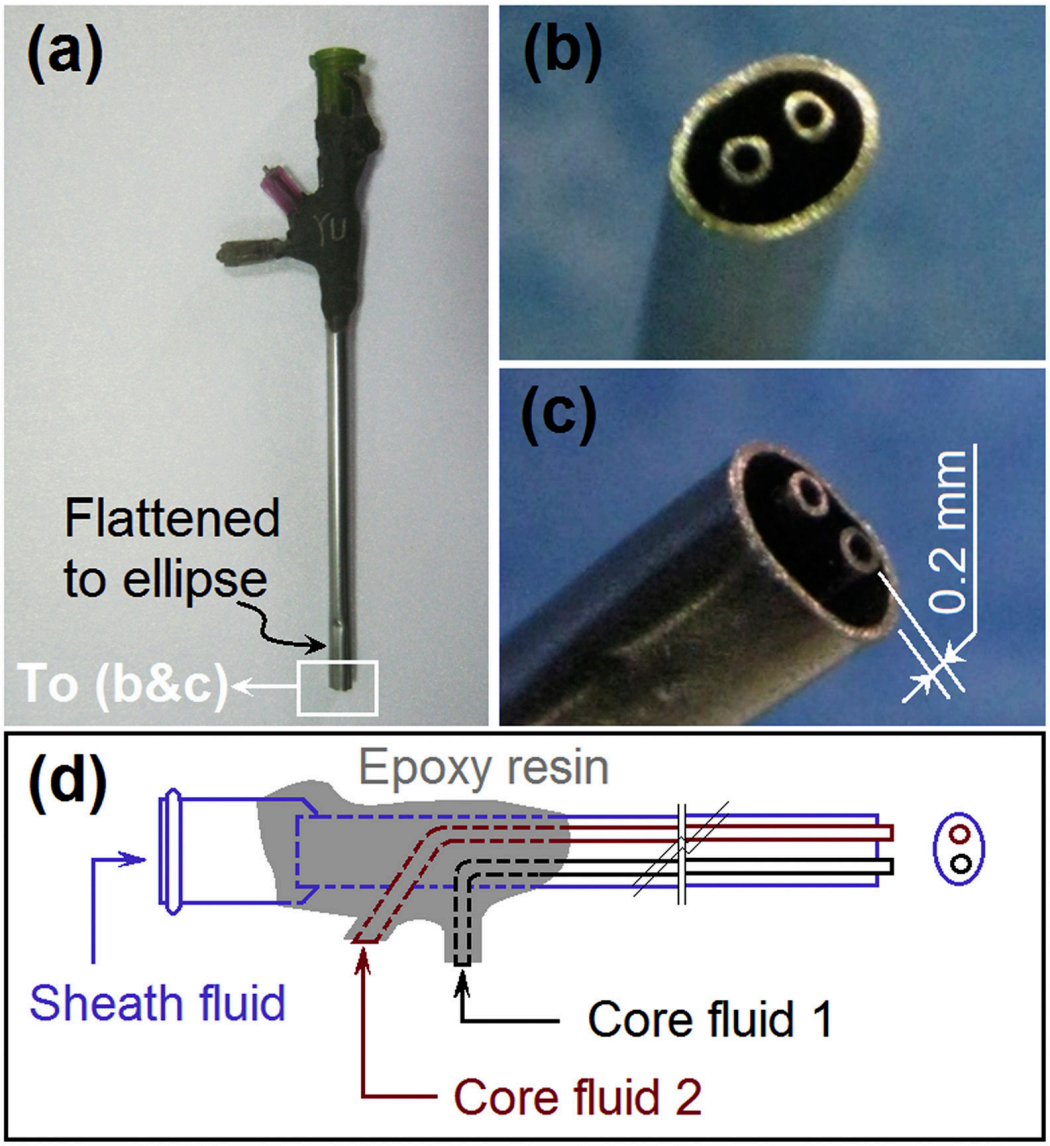
Designs of the complex spinneret for implementing trifluid electrospinning: (**a**) Digital image showing a full view of the spinneret; (**b**) front view; (**c**) side view; (**d**) diagram about organization of a structural outlet from three inlets. Reprinted from ref. [72]. Copyright (2020), with permission from Elsevier.

A spinneret with an eccentric two-nozzle configuration was utilized to prepare fibers with a double core–sheath structure. This configuration consisted of two nozzles nested within a common nozzle, which served to guide and organize the three fluids in a manner that the resulting two cores in the fiber are surrounded with a sheath (Figure 3) [72].

In conclusion, the electrospinning using a multi-nozzle spinneret enables the preparation of fibers with a core–sheath, core–double sheath or double core–sheath fibers structure, depending on the number of nozzles used. A drawback of this technology is that the control of the parameters is more complex than in single-nozzle spinneret electrospinning, particularly in terms of preparing fibers with a uniform architecture. It is for this reason that the industrial application of electrospinning using a multi-nozzle spinneret remains a challenge.

### 2.2. Single-Nozzle Spinneret Electrospinning of Emulsions

Single-nozzle spinneret electrospinning of emulsions (emulsion electrospinning) has been considered as an alternative to the coaxial electrospinning for the preparation of core–sheath fibers [46,50,55,73,74,75,76,77,78,79,80].

There are currently no data available regarding the preparation of fibers with more complex architectures, such as core–double sheath structures, by emulsion electrospinning. An advantage of the emulsion electrospinning over the coaxial one is that it is single-nozzle spinneret-based, meaning that the conventional electrospinning equipment can be used. This facilitates the straightforward transition from single-nozzle spinneret to needleless multi-jet electrospinning [74] and the potential for industrial production of core–sheath fibers. The achievements in the field of the emulsion electrospinning, as well as the main parameters altering the formation of core–sheath fibers by this technique, are discussed thoroughly in the following review articles [50,55,73]. A relatively large number of examples of polymers (natural and synthetic) that have been used for preparation of core–sheath fibers, as well as of additives added in the emulsions for providing the stability of the latter, are given in the review article by Ghosh et al. [55].

**Figure 4:**
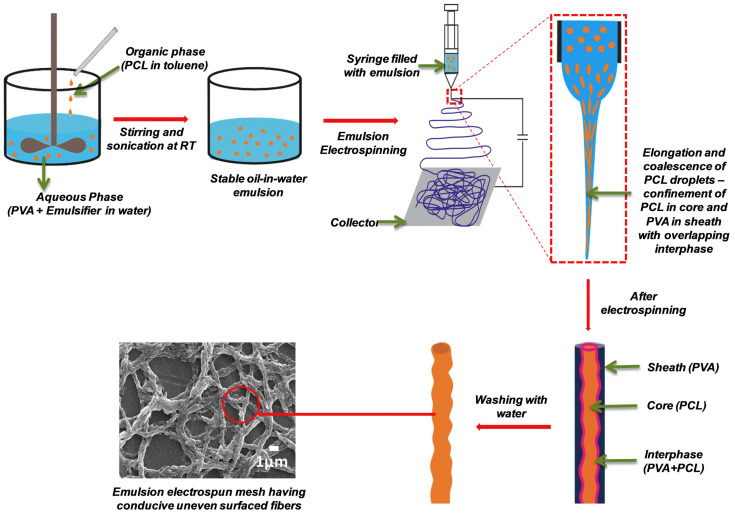
Preparation of conductive uneven surfaced mesh of PCL via emulsion electrospinning. Reprinted from ref. [80]. Copyright (2014), with permission from Elsevier.

The preparation of core–sheath fibers by electrospinning of emulsions water/oil (w/o; dispersed phase–water, and continuous phase–oil) [75,76,77] or oil/water (o/w; dispersed phase–oil, and continuous phase–water) [78,79] have been studied. For stabilization of the emulsions, stabilizers are used: emulsifiers/surfactants (e.g., Tweens, Spans, etc.), Pickering particles (e.g., nano silica, nano clay etc.), or biopolymers (e.g., soy proteins, whey protein etc.). The formation of core–sheath fibers is based on the occurrence of phase separation between the dispersed phased (the drops of the emulsion) and the continuous phase under the action of the electric field during the electrospinning process (Figure 4). In addition to the parameters that affect conventional electrospinning, such as process parameters and environmental parameters, the emulsion parameters play a primary role in the emulsion electrospinning. The emulsion parameters include: solvent nature, polymer nature and concentration, viscosity, dispersed phase volume fraction, stabilizer nature and concentration, and size of the drops of the dispersed phase [55].

The necessity to use a stabilizer, particularly when it is a synthetic surfactant, is regarded as a disadvantage of emulsion electrospinning for the fabrication of core–sheath fibers. The removal of these types of stabilizers from the fiber material is often challenging, which can subsequently result in complications pertaining to the biocompatibility of the electrospun fibers [73]. Another shortcoming of emulsion electrospinning is the necessity to consider a vast array of parameters inherent to a given emulsion in order to achieve the formation of uniform core–sheath fibers.

### 2.3. Single-Nozzle Spinneret Electrospinning of Homogeneous Blend Solutions

The electrospinning of homogeneous blend solutions using a single-nozzle spinneret has emerged as a feasible approach for preparation of composite core–sheath fibers from particular polymer-based blend systems, wherein the limitations of coaxial and emulsion electrospinning can be effectively addressed.

This approach is based on self-organization of the components in the core or sheath of the fibers as a consequence of the occurrence of phase separation of the components during the electrospinning process. Despite the extensive research on single-nozzle spinneret electrospinning of homogeneous blend solutions [3,81], there is a scarcity of studies examining the process parameters and the polymer properties on the resulting fiber architecture. Transmission electron microscopy (TEM) analyses for establishing fiber architecture are lacking in the majority of electrospinning studies on homogeneous blend solutions.

This section provides an overview of the current knowledge regarding the preparation of core–sheath(s) composite fibers from homogeneous blend solutions by single-nozzle spinneret electrospinning. It is noteworthy that there is no proposed universal mechanism for the formation of core–sheath(s) fibers by single-nozzle spinneret electrospinning of homogeneous blend solutions. The mechanism depends on the nature and properties of the used components and solvent system, and the parameters of the electrospinning process. Furthermore, as aforementioned in contrast to the coaxial and emulsion electrospinning that have been subject of numerous review articles, no review paper is available devoted to the single-nozzle spinneret electrospinning of homogeneous blend solutions. Herein, a critical overview is made on the state-of-the-art of the latter approach for preparation of core–sheath(s) fibers.

#### 2.3.1. Preparation of Core–Sheath Fibers

Depending on the nature of the used components, the pairs known that give core–sheath fibers by electrospinning of their homogeneous blend solutions are divided in this review into: (i) non-ionogenic polymer/non-ionogenic polymer or non-ionogenic polymer/low-molecular-weight substance; and (ii) non-ionogenic polymer/polyelectrolyte pair. This classification was made on the basis of the distinctive behavior of the polyelectrolytes in the presence of an electric field which is a consequence of the presence of ionizable functional groups in their structure.

##### Non-Ionogenic Polymer/Non-Ionogenic Polymer Pair or Non-Ionogenic Polymer/Low-Molecular-Weight Substance Pair

For the first time, the approach of single-nozzle spinneret electrospinning of homogeneous blend solutions nanofibers with core–sheath architecture was applied using a solution of polyaniline (PANI, 65,000 g/mol) and polystyrene (PS, 280,000 g/mol) or polycarbonate (PC, 21,900 g/mol) [PANI/polymer = 20/80 (*w*/*w*)] [56]. In the same study, it was shown that the single-nozzle spinneret electrospinning of homogeneous blend solutions of PANI and poly(methyl methacrylate) (PMMA, 120,000 g/mol) or poly(ethylene oxide) (PEO, 200,000 g/mol) does not result in the formation of core–sheath fibers. Instead, it leads to the production of fibers comprising isolated PANI domains arranged in bead-like structures. It was hypothesized that the phase morphology of the electrospun fibers depends on the molar mass of the polymers as well as the incompatibility of the polymers used. In this study, TEM analyses have been employed as the sole technique to demonstrate the core–sheath structure. Adequate analysis to ascertain the composition of the fiber core and sheath is lacking. It has been suggested that the core is PANI, and the sheath is an insulating polymer.

In their study, Wei et al. [82] examined the internal morphology of fibers prepared by single-nozzle spinneret electrospinning of homogeneous blend solutions of polybutadiene (PB) and PC. The objective of this study was to prepare nanofibers with tunable morphology, such as core–sheath or co-continuous structure, by single-nozzle spinneret electrospinning of homogeneous blend solutions. The effect of the polymer blend composition, polymer molar mass, and solvent nature on the resulting fiber morphology was evaluated. The total polymer concentration in the spinning solution at various PB/PC ratios is not specified in the report. The authors asserted that the electrospinning of polymer blend solutions has the potential to yield unique morphologies that may be used in a multitude of applications. In order to investigate the phase morphology obtained, the following parameters were varied: PB/PC weight ratio in tetrahydrofuran (THF) (90/10, 75/25, 65/35, 50/50, 35/65, 25/75 or 10/90); PC molar mass (21,900, 23,300, 27,000 or 30,600 g/mol) or PB molar mass (420,000 or 2,500,000 g/mol) at PB/PC = 75/25 or 25/75 (*w*/*w*). The prepared fibrous materials was only characterized in terms of fiber internal morphology by TEM. It was found that at PB (420,000 g/mol) content equal or higher than PC (21,900 g/mol) content, the single-nozzle spinneret electrospinning of homogeneous blend solutions of PB/PC pair in the common solvent THF fibers with co-continuous structure are obtained having interconnected nanolayers form PB and PC or strands and the nanolayers are aligned in fiber length axis direction. At an excess of PC [PB/PC = 25/75 or 10/90 (*w*/*w*)], the electrospinning of homogeneous blend solutions results in preparation of core–sheath fibers and, as stated by the authors [82], the core is PB, and the sheath is PC. The formation of core–sheath fibers is explained by the lower viscosity of PC compared to PB. The lower viscosity of the entire blend system, both in the initial state and during the rapid evaporation of the solvent, favors the polymer chains to have greater mobility, allowing PC chains to migrate, merge, and form large domains (e.g., stratified structures) so that PB and PC phases are well separated and, for example, form a core–sheath structure. In addition, the lower viscosity can provide time for PB lower molar mass phase to migrate to the surface of the resulting fiber, while the PB phase will localize in the center of the fiber, forming a core–sheath structure. An excess of PB [PB/PC > 25/75] produces fibers with a co-continuous structure rather than a core–sheath structure. This is attributed to a decrease in the ability of PC to migrate due to the higher viscosity of the polymer solution (data on the viscosity of the polymer solutions studied are lacking). It was found that the polymer molar mass alters the fiber morphology at the same ratio between PB and PC (PB/PC = 25/75). A change in morphology from core–sheath to co-continuous was observed when the molar mass of PC increases from 21,900 to 23,300 g/mol. PC is the predominant component in the blend solution at PB/PC ratio of 25/75 (*w*/*w*). An increase in the molar mass of PC results in an elevated viscosity, which effectively hinders the formation of large stratified domains and core–sheath structures. Concurrently, the mobility of PC chains is also diminished. As a result, co-continuous structures are formed at a molar mass of PC above 21,900 g/mol.

For preparation of homogeneous blend solutions that have been subjected to single-nozzle spinneret electrospinning, the following systems have also been studied: PB/PS, PMMA/PS, PB/PC, PANI/PC, and PMMA/PC [83]. The polymer pairs, polymer molar masses, and the resulting internal fiber structures are listed in Table 1. It has been found that the formation of core–sheath structures depends on both thermodynamic (interfacial tension, differences in solubility parameter of the components and their molar masses) and kinetic factors (rheological properties of the spinning solution and mobility of the components).

The incompatibility and large difference in the solubility parameter of two polymers favors the formation of well phase-separated nanofiber morphologies. The ability for preparation of such structures is governed by kinetic factors because of the rapid evaporation of the solvent during the electrospinning process. In this regard, the low molar mass of a polymer is of particular significance with respect to the formation of core–sheath structures. This outcome is attributable to the enhanced mobility of the low molecular mass component, which facilitates more effective phase separation and the development of larger domains. The rheological properties are also a determining factor in the structure of the resulting fiber. It has been proposed that the higher viscous material is localized in the fiber core, while the lower viscous material is localized in the fiber sheath.

Since the reports of Wei et al. [56,82,83], there have been several studies on the preparation of core–sheath fibers by single-nozzle spinneret electrospinning of homogeneous blend solutions comprising polymer/polymer or polymer/low-molecular-weight pairs. These studies are discussed in brief below.

For a polyacrylonitrile-poly(vinyl pyrrolidone) (PAN-PVP) pair, water vapor has been used as a phase separation inducing agent during the single-nozzle spinneret electrospinning of homogeneous blend solutions of the polymers in dimethylformamide (DMF), yielding core–sheath fibers [84]. It was found that when the electrospinning process is carried out in the absence of water vapor (at a specified humidity of 20%), phase separation did not occur, and consequently, no PAN/PVP core–sheath fibers are observed. While the experimental section of the paper indicates that the ambient humidity for electrospinning is 20% at low ambient humidity, it is notable that the authors have not specified the ambient humidity for electrospinning in the presence of water vapor. The lack of a sufficiently precise description of the conditions under which core–sheath fibers were obtained hinders the reproducibility of the results obtained. There are also no data on the diameter of the light aperture of the copper mesh used as a collector, through which water vapor passes from the vessel of water heated at 80 °C to the area where the fibers are formed. The solubility of water and dimethylformamide (DMF) in each other is high. Upon contact with the rising water vapor, the spinning jet rapidly dissolves the vapor in the jet solvent. Consequently, the water vapor prompts the water-insoluble PAN to migrate towards the DMF-rich regions within the jet core, thereby forming core–sheath nanofibers comprising a PAN core and a PVP sheath. The sheath composition was determined by X-ray photoelectron spectroscopy (XPS) and the measurement of the water contact angle. PAN fibrous material is hydrophobic (water contact angle of 128°), while PVP mat is hydrophilic (water contact angle < 5°). Mats from PAN/PVP = 1/1 or 1/2 (*w*/*w*) are hydrophilic. This indicates that the sheath of PAN/PVP fibers is enriched in PVP.

Core–sheath fibers from PVP/poly(vinylidene fluoride) (PVDF, 1 × 10^5^ g/mol; PVP, 360,000 g/mol) with superhydrophobic surface have been prepared by single-nozzle spinneret electrospinning of blend solutions using DMF/acetone = 8/2 (*w*/*w*) as a common solvent system [59]. Core–sheath fibers were prepared for all PVDF/PVP weight ratios studied (3/1, 2/1, 1/1, 1/2, and 1/3). At PVDF/PVP = 3/1, 2/1, and 1/1 (*w*/*w*), the sheath of PVDF/PVP core–sheath fibers was found to be mainly composed of PVDF, while at PVDF/PVP = 1/2 and 1/3 (*w*/*w*), they were composed of PVP. The composition of the fiber sheath was evidenced by XPS. The results obtained by XPS have been in accordance with the water contact angle data. When the PVDF content is equal to or greater than PVP, the nanofibrous mats are hydrophobic with a water contact angle value above 120°. This indicates that PVDF is mainly concentrated in the fiber sheath. For PVDF/PVP mats with high content of the hydrophilic PVP, the surface of the fibrous materials is hydrophilic, indicating that the fiber sheath is mainly composed of PVP. The results obtained suggest that core–sheath nanofibers with tailored composition of the core and sheath, as well as with favorable hydrophilic-hydrophobic behavior, can be prepared by tuning the PVDF/PVP ratio.

A methodology for the fabrication of core–sheath fibers is proposed, wherein the core is constituted by an electrospinnable, non-ionogenic polymer (PVP or PAN), while the sheath is formed by a polymer synthesized by in situ photopolymerization, either during the electrospinning process itself or in a subsequent step where the photopolymerization is conducted using an electrospun non-woven textile [85,86]. This has been attributed to the migration of a low-molecular-weight monomer and/or photoinitiator present in the spinning solution to the surface of the resulting fibers during electrospinning. The authors have not isolated the synthesized polymers, and there are no data on the macromolecular characteristics of the latter. Data on the presence of residual monomer in the electrospun fibrous materials are also lacking.

**Scheme 4:**
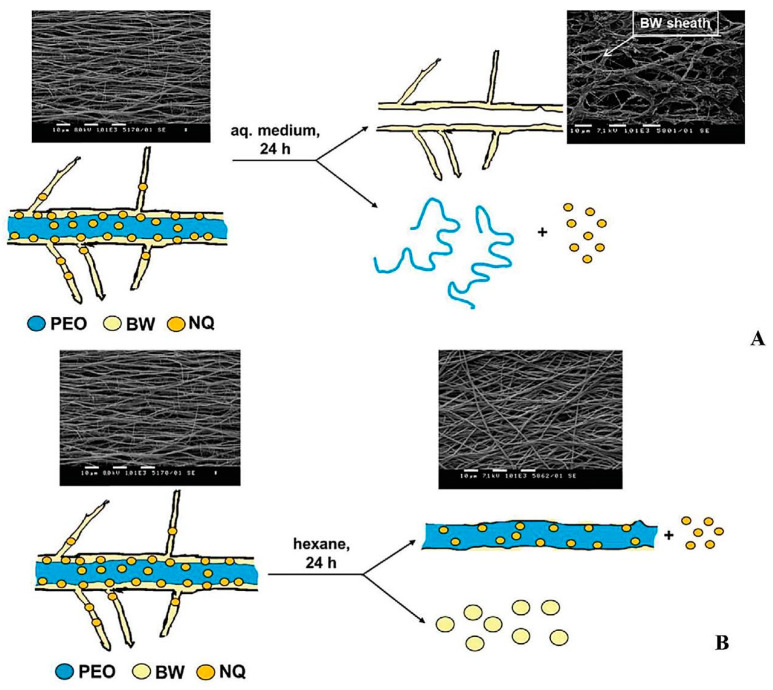
Schematically representation of fiber from [PEO/BW = 80/20]/NQ after its 24 h stay in aqueous medium (**A**) or hexane (**B**). Scanning electron microscopy (SEM) micrographs of the fibers before and after contact with aqueous medium or hexane are shown as well. Reproduced with permission from ref. [61]. Copyright (2022), John Wiley & Sons.

**Figure 5:**
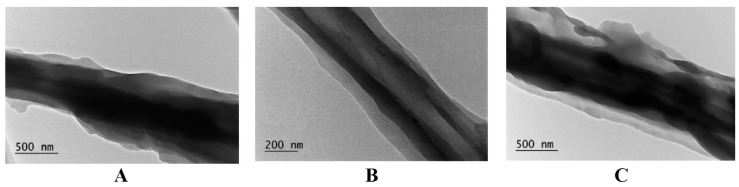
Transmission electron microscopy (TEM) micrographs of a fiber from PEO/BW = 80/20 (*w*/*w*) (**A**), PEO/BW = 70/30 (*w*/*w*) (**B**), and PEO/BW = 60/40 (*w*/*w*) (**C**). Magnification: ×10,000 (**A**,**C**) or ×20,000 (**B**). Reproduced with permission from ref. [61]. Copyright (2022), John Wiley & Sons.

The natural product beeswax (BW) is a mixture of low-molecular-weight saturated hydrocarbons, free fatty acids, free fatty alcohols, and esters of fatty acids and fatty alcohols, with myricyl palmitate (~70%) being the major component [87,88] which has hydrophobic behavior. Recently, Kyuchyuk et al. [61] demonstrated the possibility of one-step hydrophobization of the fiber surface of the hydrophilic PEO fibers by single-nozzle spinneret electrospinning of homogeneous solutions of blends of this polymer with BW in the common solvent chloroform. The beeswax used was of purity in accordance with the European Pharmacopoeia. The core–sheath structure was observed by TEM (Figure 5), and the sheath was evidenced to be BW by XPS. 5-Nitro-8-Hydroxyquinoline (NQ) was selected as a model drug to demonstrate the potential of PEO/BW fibrous materials as carriers of bioactive substances [61]. The good solubility of NQ in chloroform enabled its incorporation in the solution containing PEO and BW. The analysis by TEM showed that NQ does not alter the fiber architecture and they are core–sheath ones. To demonstrate that the core of PEO/BW/NQ fibers consists of PEO and the sheath of BW, selective extraction of PEO or BW in aqueous medium or in hexane, respectively, was performed (Scheme 4). In aqueous media, PEO and NQ dissolve and BW remains insoluble. It was demonstrated that after a 24 h stay in aqueous medium, PEO and BW leave the fibrous structure of the mats and dissolve in the aqueous medium (Scheme 4A).

SEM observation of the fragmented structures obtained by soaking the PEO/BW/NQ mat in aqueous medium for 24 h has shown that they resemble a fiber cut along its length (Scheme 4A). To further demonstrate the core–sheath structure, BW was extracted by soaking the PEO/BW/NQ mat in hexane for 24 h (hexane is a good solvent for BW, but does not dissolve PEO and NQ). As seen from the SEM micrograph presented in Scheme 4B, after the extraction, the mat is composed of uniform cylindrical fibers. The latter are soluble in aqueous medium. The extraction results (in aqueous medium or in hexane) have proven that the PEO/BW/NQ fibers are core–sheath ones, having of a PEO core and a BW sheath. The formation of PEO/BW or PEO/BW/NQ fibers with core–sheath architecture has been attributed to self-organization of BW molecules on the fibers surface during the electrospinning process, driven by the incompatibility between PEO and BW, as well as by the air hydrophobicity. Furthermore, the distinction in molar mass between PEO and BW is a significant factor. BW, as a mixture of low-molecular-weight substances, exhibits higher mobility compared to PEO in the fiber-forming process during the electrospinning, whereby it forms the fiber sheath, while PEO is responsible for the formation of the fiber core. The preparation of core–sheath fibers from the PEO/BW system by means of single-nozzle spinneret electrospinning of homogeneous blend solutions of the components is attractive because it enables the one-pot hydrophobization of the PEO fibers surface. This is important for expanding the applicability of hydrophilic PEO fibrous materials. PEO is regarded as an invaluable and prospective polymer component in a multitude of pharmaceutical and cosmetic formulations.

##### Non-Ionogenic Polymer/Polyelectrolyte Pair

Polyelectrolytes are a class of polymers that contain functional groups along their chain, such as polyethyleneimine, or as side groups [e.g., poly(acrylic acid), chitosan, sodium alginate] that are capable of ionization in aqueous medium. Depending on their charge, the polyelectrolytes are divided into polycations (e.g., chitosan, polyethyleneimine), polyanions [e.g., poly(acrylic acid), sodium alginate], or polyampholytes/polyzwitterions (e.g., *N*-carboxyethylchitosan, peptides). Moreover, a number of polyelectrolytes have been demonstrated to possess biological activity. The biological activity of polyelectrolytes renders them promising candidates for the development of novel polymer materials for cell and tissue engineering, as well as in biomedical applications (e.g., drug carriers, wound dressings) [89]. Polyelectrolytes of natural origin, including chitosan (CS), sodium alginate (SA), and hyaluronic acid (HA), are of particular interest due to their biodegradability.

The preparation of composite core–sheath fibers is an intriguing approach to impart bioactivity to electrospinnable, water-soluble or water-insoluble, biologically inert non-ionogenic polymers. It has been found that, in aqueous solution, it is impossible to reach a concentration of polyelectrolyte at which efficient entanglement of its macromolecules occurs. This is due to the presence of ionic groups in the structure of polyelectrolytes and, therefore, the presence of repulsive forces between the charges [90,91]. This is the reason for the impossibility for preparation of fibers by electrospinning of an aqueous solution of a polyelectrolyte.

The most studied approach for preparation of polyelectrolyte-containing fibers is the electrospinning of an aqueous homogeneous blend solution of the polyelectrolyte and an electrospinnable, synthetic, non-ionogenic, water-soluble polymer [45,92,93,94]. Another approach is to use a solvent such as trifluoroacetic acid (TFA). This allows the preparation of composite fibers from polyelectrolyte and biocompatible, biodegradable, water-insoluble polyester [45,95]. There are studies on the possibility of forming composite core–sheath fibers from polyelectrolyte/water-soluble polymer and polyelectrolyte/water-insoluble polymer, but they are episodic and not systematic. They are briefly discussed below.

A large number of research groups have used the approach of electrospinning of an aqueous solution of a polyelectrolyte and a synthetic, non-ionogenic, electrospinnable polymer; but few have evaluated the architecture of the resulting fibers by TEM. The initial reports on electric field-driven enrichment of the surface of non-ionogenic polymer/polyelectrolyte fibers with polyelectrolyte chains have been for the following pairs: PEO/PEO-peptide conjugate and PCL/PCL-*b*-poly[(2-dimethylamino) ethyl methacrylate] [96], subjected to single-nozzle spinneret electrospinning of their homogeneous blend solution in a common solvent. The enrichment with a polyelectrolyte has been evidenced by XPS. However, these reports lack observations by TEM to determine whether the fibers have a core–sheath structure.

In 2009, the first report was published on preparation of core–sheath fibers from the non-ionogenic/polyelectrolyte system by single-nozzle spinneret electrospinning of homogeneous solutions of the components [97]. Using PEO as a non-ionogenic polymer and chitosan as a polyelectrolyte component Zhang et al. [97] demonstrated that the single-nozzle spinneret electrospinning of aqueous homogeneous solutions of both components in deionized water (in the absence of glacial acetic acid in the case of chitosan oligomers; or with addition of glacial acetic acid when using a high molar mass chitosan) results in preparation of core–sheath fibers. A positive high voltage supply source was applied. The effect of PEO/chitosan ratio, chitosan molar mass (3000, 10,000, 50,000 or 200,000 g/mol) and the temperature of the electrospinning process on the resulting fibers’ structure was evaluated. PEO with a molar mass of 900,000 g/mol was used. It was found that for the same molar mass of chitosan (10,000 g/mol), increasing the polycation content in the spinning solution results in core–sheath fibers with a smaller core-to-fiber diameter ratio. This was attributed to a thicker chitosan sheath in PEO/chitosan fibers as the polyelectrolyte content increases. Regarding the effect of chitosan molar mass on the core–sheath structure of the fibers, it was demonstrated that the fibers prepared using chitosan with the lowest molar mass (3000 g/mol) have the thickest chitosan sheath. This is attributed to chitosan chain mobility. The low molar mass (3000 g/mol) of chitosan facilitates phase separation in the blend system, allowing for the formation of a small core-to-fiber diameter ratio due to the high mobility of the chitosan chains. As the solvent evaporates, PEO has the potential to crystallize within the fiber. Additionally, it is known that PEO is prone to crystallization and possesses a relatively low melting point, ranging from 66 to 70 °C. The authors stated that at higher temperatures (equal or higher than 70 °C) core–sheath fibers are not formed, but instead become monolithic fibers composed of a blend of PEO and chitosan macromolecules. The fiber sheath composition was assessed by TEM equipped with energy dispersive spectroscopy (EDS) by the presence of nitrogen on the fiber surface. The presence of nitrogen (only present in chitosan structure) in the EDS spectrum indicates that PEO/chitosan core–sheath fibers consist of a PEO core and a chitosan sheath.

Following this work, the research group of Nie et al. published several reports addressing the possibility for preparation of core–sheath fibers from a water-soluble non-ionogenic polymer/polyelectrolyte blend using water as the common solvent for the components [98,99,100]. The used polymer pairs, as well as the composition of the core and the sheath of the resulting core–sheath fibers, are listed in Table 2. They used positive high-voltage power source for electrospinning, and the sheath composition was determined by XPS. It was demonstrated that the composition of the core and the sheath of the core–sheath fibers from non-ionogenic polymer/polyelectrolyte depends on the polyelectrolyte nature. Using the polycation CS, it was shown that the resulting core–sheath fibers are composed of a core of the non-ionogenic polymer (PVA [98] or PEO [99]) and CS sheath. The use of a polyanion (HA or SA) led to preparation of core–sheath fibers having a core of the polyanion and a sheath of the non-ionogenic polymer (PVP [98], PVA [98], or PEO [98,100]). The formation of core–sheath fibers during the electrospinning of homogeneous blend solutions of non-ionogenic polymer/polyelectrolyte pair has been proposed to be driven by the electric field. In non-ionogenic polymer/non-ionogenic polymer solutions, phase separation between the components in the liquid spinning jet occurs during the solvent evaporation when the concentration of the components reaches a threshold level, whereby the diminished solvation effect of the solvent is no longer sufficient to protect the polymers in the blend from phase separation. The phase separation process is terminated when the concentration of polymers reaches a level at which their mobility is no longer permitted. In the case of solutions of non-ionogenic polymer/non-ionogenic polymer systems, the range of polymer concentrations at which phase separation occurs during electrospinning is relatively narrow, with a correspondingly short phase separation time due to the rapid solvent evaporation. Accordingly, for the preparation of fibers with core–sheath architecture, the polymer mobility is of crucial importance. In non-ionogenic/polyelectrolyte solutions, the applied electric field facilitates enhanced polyelectrolyte chain mobility.

Furthermore, the electric field has been demonstrated to expand the phase separation window in the electrospinning process of blend polyelectrolyte-based systems. In this case, phase separation occurs when an electric field is applied and can be stopped as the jet solidifies during the electrospinning process.

**Figure 6:**
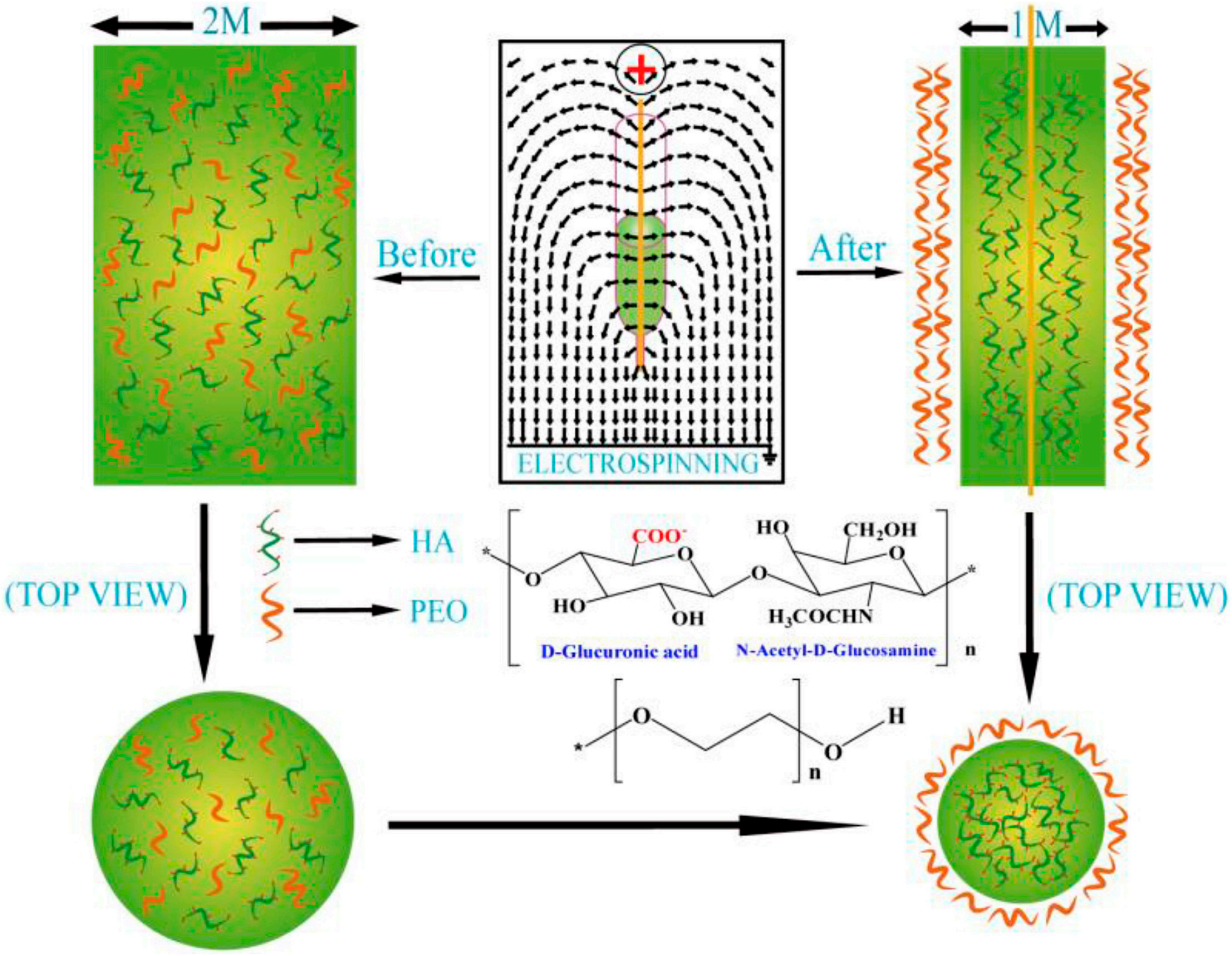
Schematic diagram of preparation of PEO/HA core–shell nanofibers. Reprinted from ref. [100]. Copyright (2016), with permission from Elsevier.

For PEO/HA pair, single-nozzle spinneret electrospinning of homogeneous blend solutions of the components using water as a common solvent and a highly positive electric potential has been found to result in core–sheath fibers, with a core composed of the polyelectrolyte and a sheath composed of the non-ionogenic polymer (Figure 6) [100]. An explanation has been found in HA polyanionic behavior. It has been hypothesized that under the action of the positive electric potential utilized in the electrospinning, HA macromolecules would exhibit a movement in a direction opposite to the electric field lines, thereby inducing phase separation with PEO and the formation of core–sheath fibers comprising a core of HA and a sheath of PEO. PEO with molar mass of 900,000 g/mol and HA with molar mass of 8700 g/mol were used. The ratio between the two components was varied (PEO/HA = 3/1, 1/1, or 1/3). TEM analysis revealed that core-to-fiber diameter ratio shows an increasing trend with increasing HA content. Therefore, with increasing HA content, the core diameter increases. XPS analysis demonstrated that nitrogen content on the surface of core–sheath PEO/HA fibers is merely 0.78% (in HA powder, nitrogen content is 4.8%). This is close to that of PEO non-woven textile (0%), indicating that the predominant composition of the sheath in PEO/HA nanofibers is PEO, with the majority of HA present in the core of the fibers.

Using bovine serum albumin (BSA) as a polyelectrolyte, Won et al. [57] prepared nanofibers with core–sheath architecture by single-nozzle spinneret electrospinning of PVA and BSA using water as a common solvent. The core was of PVA, and the sheath was of BSA. BSA is a biopolymer that contains both carboxyl and amino groups [101]. For the electrospinning, positive high voltage was used. The results of the TEM analysis indicate that at PVA/BSA weight ratios of 9/1, 7/3, and 5/5, the fibers exhibit a core–sheath structure, with an increase in the thickness of the sheath as the BSA content in the fiber composition rises. The authors suggested that in the case of electrospinning of homogeneous PVA/BSA blend solutions, the formation of the core–sheath structure may be attributed to phase separation in the polymer blends at an optimal applied voltage. This phenomenon can be attributed to the fact that the lower molar mass BSA is capable of undergoing a strong ionization process as a result of the applied voltage during the electrospinning process. This leads to the rearrangement of the BSA in the outer layer of the composite nanofibers, thereby forming a core–sheath structure. The presence of a nitrogen atom on the fiber surface, as detected by XPS analysis, indicates the presence of a BSA sheath. The intensity of this nitrogen peak increases with increasing BSA content in the PVA/BSA composite fibers. TEM analyses of fibers prepared at PVA/BSA = 5/5 at 15, 22, or 29 kV indicate that the use of an inappropriate high voltage does not result in the formation of core–sheath fibers, and for this polymer pair, the optimum voltage was found to be 22 kV.

As aforementioned, the second approach for preparation of polyelectrolyte-containing composite fibers is by electrospinning of a solution thereof in the presence of a biodegradable and biocompatible, non-ionogenic, electrospinnable polyester. Since the polyester is water-insoluble, it is necessary to find a suitable solvent system for preparation of a homogeneous blend solution from the polyester/polyelectrolyte system. For example, using TFA as a common solvent, Xu et al. [102] prepared fibers with complex nanoscale surface topography by single-nozzle spinneret electrospinning of homogeneous blend solutions of PLA and CS. The experimental section does not specify whether poly(L-lactide), poly(D-lactide) or a copolymer of L- and D-lactide were used. This is relevant to the crystallizability of the polylactide: poly(L-lactide) and poly(D-lactide) are capable of crystallization, while poly(D,L-lactide) is an amorphous polymer.

**Figure 7:**
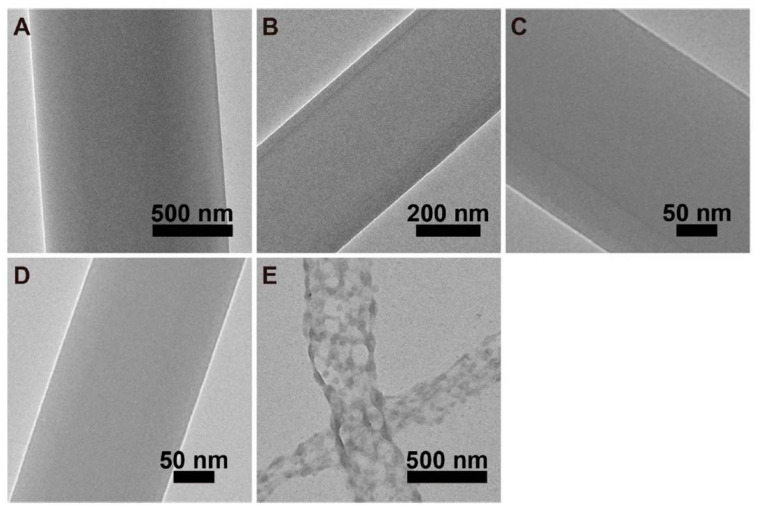
TEM images of electrospun PLA/CS. (**A**) PLA; (**B**) PLA /CS 70:30; (**C**) PLA /CS 10:90; (**D**) CS; (**E**) PLA/CS70:30, 35 °C, etched by dichloromethane. Compared to neat PLA or CS, all composite PLA/CS fibers display a core–shell prepared under room temperature. Sharp contrast of bright and dark regions in the TEM images displays the morphology of a hollow interior but an external continuous “cellular” structure, and proves the distribution of binary polymer PLA and CS in the island-like fibers. Reproduced with permission from ref. [102]. Copyright (2017), American Chemical Society.

The molar mass of CS used was also not provided. It was not specified whether the applied high voltage was positive or negative. Depending on the temperature at which the electrospinning was conducted, nanofibers with core–sheath or island-like architecture were obtained. Electrospinning at 25 °C and CS/PLA weight ratio from 90/10 to 10/90 results in preparation of core–sheath fibers and smooth surface topography. The electrospinning at higher temperature of 35, 45, 50, 55, or 60 °C (a description of the methodology employed to maintain these temperatures during the electrospinning process is lacking in the experimental section) leads to the preparation of fibers with a rough, island-like surface topography. TEM micrographs of core–sheath PLA/CS nanofibers are shown in Figure 7A–D. Applying selective dissolution of PLA from island-like fibers in dichloromethane, it was found by TEM that the morphology of the resulting structure is a hollow interior with an outer continuous cellular structure (Figure 7E). It has been stated that this is an indication that the “islands” are made of CS and the “sea” is composed of PLA. For core–sheath fibers, the core is PLA and the sheath is CS, as evidenced by XPS. Similarly to the PEO/CS system [97], the formation of core–sheath fibers from PLA/CS has been attributed to phase separation resulting from the solvent evaporation during the electrospinning process [102]. Another crucial factor has been identified as the preferential migration of the positively charged chitosan chains to the surface of the charged spinning jet, and thus to the fiber surface during the electrospinning process. Increasing the electrospinning temperature increases the evaporation rate of the solvent, and some of the CS chains have no time to migrate to the spinning jet surface, so there is not enough time for the CS component to cover the entire fiber surface and intermittent islands are formed. This has been the explanation for the preparation of island-like fibers when electrospinning is performed at a higher temperature.

In conclusion, the use of polyelectrolytes enables the obtaining of composite core–sheath fibers comprising a non-ionogenic polymer/polyelectrolyte pair in a single step, without the need for an auxiliary coaxial device and in the absence of stabilizing agents, by conventional single-nozzle spinneret electrospinning of homogeneous solutions of the components in a common solvent. This is attributed to the ability of the polyelectrolytes to become electrically charged under the action of an electric field. Depending on the charge of the applied high voltage (positive or negative) and the polyelectrolyte nature (polycation or polyanion), core–sheath fibers can be prepared with a tunable core and sheath composition: a core of a non-ionogenic polymer and a sheath of a polyelectrolyte, or a core of a polyelectrolyte and a sheath of a non-ionogenic polymer. Having in mind the biological activity of a significant proportion of the polyelectrolytes, it is reasonable to hypothesize that the core–sheath fibers based on polyelectrolytes would display intriguing properties with regard to biomedical applications. A summary of the current knowledge regarding the potential biomedical applications of these fibers can be found in Section 3 of this review.

#### 2.3.2. Preparation of Core–Double Sheath Fibers

Kyuchyuk et al. [62,63] were the first to demonstrate the feasibility of preparing core–double sheath fibers by single-nozzle spinneret electrospinning of homogeneous blend solutions comprising the constituents of a core and two sheaths. This has been achieved using homogeneous blend solutions of PEO, biocompatible and biodegradable aliphatic polyester, and BW. PLA, PCL, poly(D,L-lactide-*co*-glycolide) (PLAGA), poly(butylene succinate) and PHB have been used as the aliphatic polyester. The use of poly(L-lactide) with molar mass of 259,000 g/mol (PLA259k), which is lower than that of PEO (600,000 g/mol; PEO600k) and higher than that of the low-molecular-weight substances in BW composition, enables the preparation of core–double sheath fibers at all of the studied component weight ratios [62]. The fibers are composed of PEO core, PLA inner sheath, and BW outer sheath, as evidenced by the performed XPS and selective extraction of BW and PEO in hexane and aqueous medium, respectively.

**Scheme 5:**
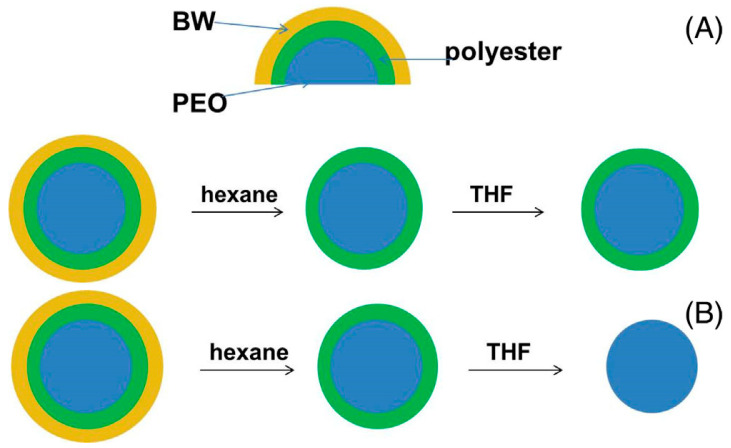
Consecutive selective extraction of the outer sheath (with hexane) and the inner sheath (with THF) of core–double sheath fibers: polyesters insoluble in THF (**A**) and polyesters soluble in THF (**B**). BW, beeswax; PEO, poly(ethylene oxide); THF, tetrahydrofuran. Reproduced with permission from ref. [63]. Copyright (2024), John Wiley & Sons.

The formation of this complex architecture has been attributed to the difference in the components’ molar masses, and, consequently, to their different ability to migrate to the surface of the spinning jet. The proposed approach to produce fibers with a core–double sheath architecture, as discussed in [62], has been validated by replacing PLA in the PEO/PLA/BW system with an alternative biocompatible and biodegradable aliphatic polyester, such as PCL, PLAGA, PBS, or PHB [63]. TEM analysis has revealed that the replacement of PLA with another polyester does not result in any alteration to the architecture of the fibers, being a core–double sheath one [63]. The impact of the molar mass ratio of PEO to the polyester on the composition of the core and inner and outer sheaths has been also assessed. A novel approach was employed, whereby a consecutive selective extraction of the outer and inner sheaths was conducted using hexane and THF, respectively, as the solvent (Scheme 5). The solubility tests of BW pellets, PEO mats, and polyester mats have shown that hexane is a good solvent of BW, and does not dissolve PEO600k and the used polyesters. This has enabled the selective extraction of BW from PEO600k/polyester/BW fibers by immersion of the fibrous materials in hexane, resulting in the removal of BW outer fiber sheath (Scheme 5A,B). PEO mats are insoluble in THF. Concerning the solubility in THF, the used polyesters are divided into THF-insoluble polyesters (PBS and PHB), and THF-soluble polyesters (PCL, PLAGA, or PLA). For THF-insoluble polyesters (PBS and PHB), the extraction in THF does not dissolve the inner polyester sheath of the fibers, and core–sheath fibers are observed by TEM (Scheme 5A). For THF-soluble polyesters, the extraction in this solvent resulted in dissolution of the inner polyester sheath, and monolithic fibers composed of the PEO core of the original fibers are observed by TEM (Scheme 5B). The application of the consecutive extraction has revealed that fibers with a well-differentiated PEO core, polyester inner sheath, and BW outer sheath can be obtained with a polyester molar mass lower than that of PEO. For mats that contain a THF-soluble polyester (PCL, PLAGA, or PLA) the experimentally determined weight loss after treatment with THF is very close to the theoretical one.

**Figure 8:**
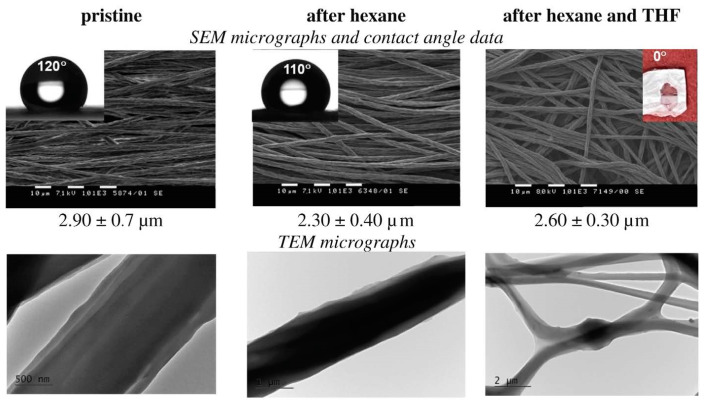
Scanning electron microscopy (SEM) (upper row) and transmission electron microscopy (TEM) (lower row) micrographs of a mat and of a single fiber, respectively, from PEO600k/PLA259k/BW fibrous materials before (left column) and after extraction with hexane (middle column), and with hexane followed by extraction with tetrahydrofuran (THF) (right column). The mean fiber diameter values are given below each SEM image; inset: water contact angle values. Magnification of the SEM micrographs: ×1000. Magnification of the TEM micrographs: ×10,000 (pristine), ×5000 (after extraction with hexane), and ×2500 (after extraction with hexane followed by extraction with THF). Reproduced with permission from ref. [63]. Copyright (2024), John Wiley & Sons.

As seen from Figure 8, PEO600k/PLA259k/BW mats, which have passed the consecutive extraction with hexane and THF, respectively, are hydrophilic with water contact angle of 0°, evidencing that they are composed of monolithic fibers derived from the PEO core of the pristine fibers. Similar results have been obtained for the core–double sheath fibers from PEO600k/PLAGA/BW and PEO600k/PCL/BW. For polyesters with a molar mass higher than PEO, it has been found that there are polyester macromolecules in the PEO core and PEO chains in the polyester inner sheath [63]. This has been attributed to the restricted mobility of the polyester chains towards the surface of the forming fiber during the electrospinning process, which results in some polyester chains becoming entrapped within the fiber core.

To assess the feasibility of targeting the deposition of hydrophilic or hydrophobic substances during the single-nozzle spinneret electrospinning of homogeneous blend solutions of PEO600k/PLA259k/BW in either the hydrophilic core or hydrophobic sheath, hydrophilic ZnO or hydrophobic ZnO(Si), respectively, have been used as model contrast agents [63].

**Figure 9:**
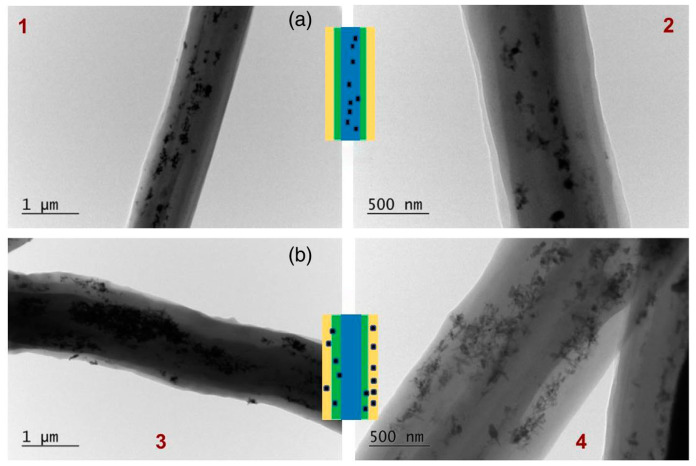
Transmission electron microscopy micrographs of a fiber from PEO600k/PLA259k/BW/ZnO (**1** and **2**) or PEO600k/PLA259k/BW/ZnO (Si) (**3** and **4**); magnifications ×5000 (**1** and **3**) or ×10,000 (**2** and **3**). Schematic representation of the preferential location of ZnO (**a**) or ZnO(Si) (**b**) in the fiber structure: PEO600k core (blue); PLA259k inner sheath (green); BW outer sheath (yellow); nanosized ZnO or ZnO(Si) (black symbols). Reproduced with permission from ref. [63]. Copyright (2024), John Wiley & Sons.

The aforementioned agents were incorporated into homogeneous blend PEO600k/PLA259k/BW solutions, and the suspensions obtained were subsequently processed via single-nozzle spinneret electrospinning. The presence of ZnO or ZnO(Si) does not impede the formation of the core–double sheath architecture (Figure 9). The localization of zinc oxide within the fiber is found to depend on the type of zinc oxide used. The hydrophilic ZnO is preferentially detected in the hydrophilic PEO core of the fiber (Figure 9(a1,a2)), while the hydrophobic ZnO(Si) is in the hydrophobic fiber sheaths (Figure 9(b3,b4)). Therefore, ZnO and ZnO(Si) are appropriate for use as model contrast agents for the visualization of the feasibility of targeted one-pot localization of a hydrophilic or hydrophobic substance during the single-nozzle spinneret electrospinning of PEO600k/PLA259k/BW homogeneous blend solutions in the core or sheaths, respectively.

It has been demonstrated that the fibers architecture and polyester nature alter the release of the model drug NQ [63]. The juxtaposition of NQ-released amount from the following mats, PEO600k/BW/NQ (core–sheath fibers), PEO600k/PCL/BW/NQ (core-double sheath fibers), and PEO600k/PLA259k/BW/NQ (core-double sheath fibers), has revealed that it decreases in the order PEO600k/BW/NQ > PEO600k/PCL/BW/NQ > PEO600k/PLA259k/BW/NQ. This indicates that the existence of a polyester inner sheath in the core–double sheath fibers from PEO600k/PCL/BW/NQ and PEO600k/PLA259k/BW/NQ results in retardation of NQ release, and a more sustained release has been registered in the case of PEO600k/PLA259k/BW/NQ mats as compared to PEO600k/PCL/BW/NQ mats. It has been assumed that the more sustained NQ release in the case of PLA inner sheath is due to the higher molar mass of this polyester (ca. 259,000 g/mol) as compared to PCL molar mass (ca. 69,000 g/mol).

From the literature survey, it can be concluded that the formation of core–sheath fiber(s) by single-nozzle spinneret electrospinning of homogeneous blend solutions, as well as the composition of the core and sheath(s), depends on the nature of the components used, their molar masses, the solvent system used, and the parameters of the electrospinning process.

## 3. Potential Applications of Core–Sheath(s) Fibers Prepared by Single-Nozzle Spinneret Electrospinning of Emulsions and Homogeneous Blend Solutions

The preparation of core–sheath fibers using single-nozzle spinneret electrospinning of emulsions and homogeneous blend solutions is a straightforward process that provides a superior platform for systematic applied research.

**Figure 10:**
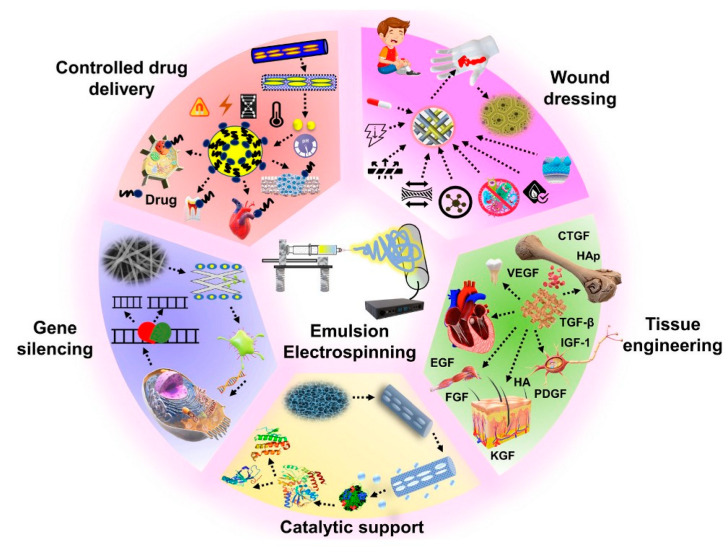
Potential applications of emulsion electrospun PCL matrices. Reproduced with permission from ref. [55]. Copyright (2023), MDPI.

The emulsion electrospinning has been a widely applied laboratory technique for fabrication of a non-woven textile comprising core–sheath fibers with tailored properties for specific applications. Particular examples can be found in the review article by Ghosh et al. [55] (Figure 10). With respect to prospective applications of non-woven textile composed of core–sheath(s) fibers prepared by single-nozzle spinneret electrospinning of homogeneous blend solutions, research in this field continues to be in its infancy. In this section, the preliminary research steps that have been undertaken in this field are delineated.

### 3.1. Biomedical Applications

The evaluation of the behavior of PEO/HA nanofibrous membranes composed of core–sheath fibers with an HA core and a PEO sheath, as prepared by single-nozzle spinneret electrospinning of homogeneous blend solutions of the components, in contact with mouse L929 fibroblast cells has shown that the mats are biocompatible and can find application in the tissue regeneration [100].

**Figure 11:**
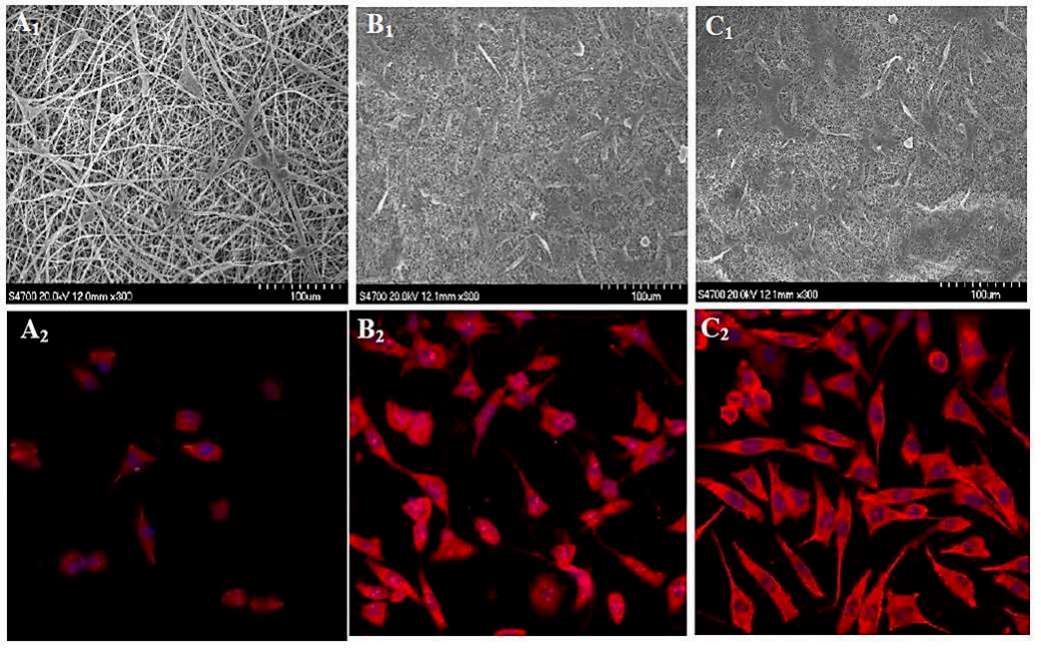
SEM and LSCM micrographs (× 400) of cells grown and attached for 48 h on different samples, stained with DAPI and TRITC-phalloidin. (**A**) pure PLA (**A_1_**,**A_2_**); (**B**) PLA/CS 70:30 with core–shell structure (**B_1_**,**B_2_**); (**C**) PLA/CS 70:30 with island structure (**C_1_**,**C_2_**). Reproduced with permission from ref. [102]. Copyright (2017), American Chemical Society.

The behavior of PLA/CS fibers having different surface topography, whether core–sheath fibers with PLA core and CS sheath, or “island-like” fibers with CS “islands” on the fiber surface (prepared by single-nozzle spinneret electrospinning of PLA/CS homogeneous blend solutions), in contact with mouse preosteoblast MC3T3-E1 cells has been studied [102]. As shown in Figure 11, the quantity of the adhered cells on PLA/CS fibers with core–sheath or “island-like” architectures is greater than that observed on PLA mats (control mats). Furthermore, a more pronounced spreading morphology of the cells has been observed in both types of PLA/CS composite mats.

A comparison of the two types of PLA/CS fiber architectures has revealed that cells exhibit enhanced adhesion (as evidenced by a higher number of adherent cells and a more pronounced spreading morphology) when in contact with island-like fibers. This has been attributed to the combination of the presence of chitosan on the fiber surface and the rough surface topography of the island-like fibers. It has been suggested that the synergistic effect of these two factors may have a beneficial impact on cell adhesion and proliferation.

NQ possesses antibacterial and anticancer activity [103,104,105,106]. In order to demonstrate the potential applications of fibrous materials from PEO/BW/NQ (core–sheath fibers), PEO/PLA/BW/NQ (core–double sheath fibers), or PEO/PCL/BW/NQ (core–double sheath fibers) prepared by single-nozzle spinneret electrospinning of homogeneous blend solutions of the components, in vitro studies have been conducted in the biomedical practice on their behavior in contact with pathogenic microorganisms and/or cancer cells [61,62,63]. The antibacterial activity has been assessed by determination of the inhibition zones of fibrous disk samples against the Gram-positive bacteria *Staphylococcus aureus* and Gram-negative bacteria *Escherichia coli*, *Pseudomonas aeruginosa*, and *Bacillus cereus*, and their antifungal activity has been assessed against *Candida albicans*.

**Figure 12:**
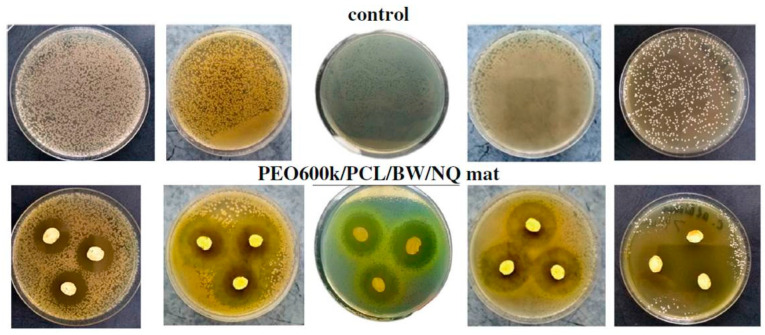
Digital images of the inhibition zones registered after a 24 h contact of PEO600k/PCL/BW/NQ mats with *Bacillus cereus*, *Staphylococcus aureus*, *Pseudomonas aeruginosa*, and *Escherichia coli*, and after a 48 h contact with *Candida albicans* (lower row). For comparison, Petri dishes with grown microorganisms in the absence of fibrous material are presented (upper row). Reproduced with permission from ref. [63]. Copyright (2024), John Wiley & Sons.

**Figure 13:**
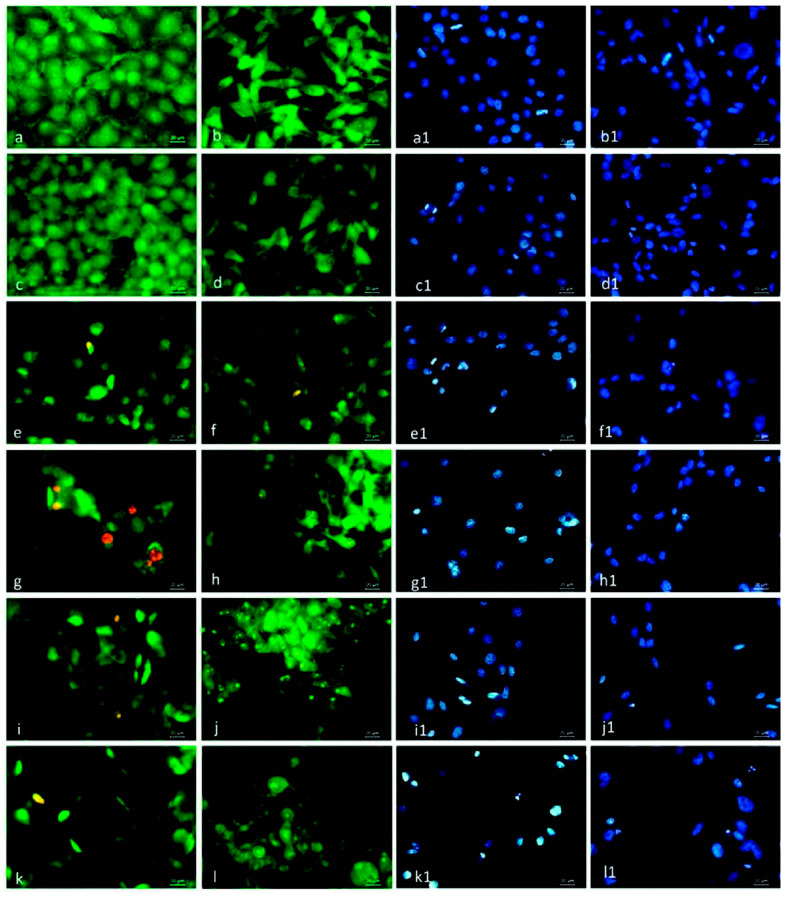
Fluorescence micrographs of AO/EtBr double-stained HeLa (**a**,**c**,**e**,**g**,**i**,**k**) and SH-4 (**b**,**d**,**f**,**h**,**j**,**l**), and DAPI-stained HeLa (**a1**,**c1**,**e1**,**g1**,**i1**,**k1**) and SH-4 (**b1**,**d1**,**f1**,**h1**,**j1**,**l1**) incubated in the presence of different fibrous mats or NQ solution for 24 h. Concentration of NQ in the mats or solution = 16 μM/L. Control: Untreated HeLa (**a**,**a1**) or SH-4 (**b**,**b1**) cells. Fibrous mats: PEO/BW = 70/30 (**c**,**d**,**c1**,**d1**), [PEO/BW = 70/30]/NQ (**e**,**f**,**e1**,**f1**), [PEO/BW = 60/40]/NQ (**g**,**h**,**g1**,**h1**), and [PEO/BW = 80/20]/NQ (**i**,**j**,**i1**,**j1**); NQ solution (**k**,**l**,**k1**,**l1**). Magnification × 300; scale bar = 20 μm. Reproduced with permission from ref. [61]. Copyright (2022), John Wiley & Sons.

The results have demonstrated that the mats composed of core–sheath(s) fibers from PEO/BW/NQ, PEO/PLA/BW/NQ, or PEO/PCL/BW/NQ exhibit antibacterial activity (Figure 12), suggesting potential applications in the wound management. The assignments of PEO/BW/NQ fibrous materials to the cancer cell lines HeLa (cervical adenocarcinoma) and SH-4 (human melanoma), as well as the normal cell line BJ (normal human skin fibroblasts), have been evaluated by MTT test [61]. The results indicate that the PEO/BW/NQ mats exhibit a detrimental impact on the viability of cancer HeLa and SH-4 cancer cells, with HeLa cells exhibiting the most pronounced decline in viability. Regarding the normal BJ human cells, the results have shown that PEO/BW/NQ fibrous materials are much less cytotoxic. This indicates that PEO/BW/NQ fibrous materials have a selective activity: they significantly reduce the viability of cancer cells while being biocompatible with normal cells. Two staining methods, intravital staining with acridine orange/ethidium bromide (AO/EtBr) and 4′,6-diamidino-2-phenylindole (DAPI) staining, have been used to ascertain whether the anticancer activity of NQ-containing fiber mats and NQ solution against HeLa and SH-4 cancer cells is associated with apoptosis induction in cancer cells (Figure 13) [61]. The results indicate that the integration of NQ into PEO/BW/NQ fibrous materials does not affect the anticancer efficacy of the drug, and the latter continues to exert its anticancer activity against human HeLa and SH-4 cancer cells through induction of programmed cell death (apoptosis).

### 3.2. Agricultural Applications

**Figure 14:**
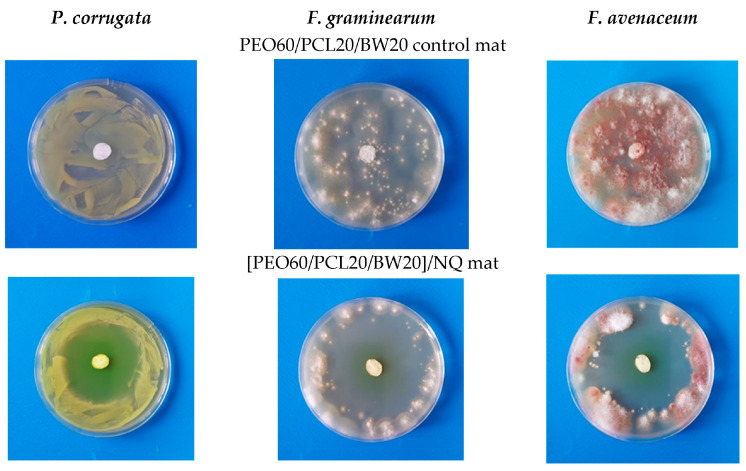
Digital images of the inhibition zones after contact of PEO60/PCL20/BW20/NQ mats with phytopathogenic microorganisms (lower row). Digital images of blank control PEO600k/PCL/BW mat after contact with the phytopathogenic microorganisms are presented for comparison (upper row). Left column: *P. corrugata*; middle column: *F. graminearum*; right column: *F. avenaceum*. Reproduced with permission from ref. [108]. Copyright (2023), MDPI.

NQ and 5-chloro-7-iodo-8-hydroxyquinoline (CQ) have antibacterial and antifungal activity against phytopathogenic microorganisms [107]. For this reason, mats consisting of PEO/BW core–sheath or PEO/polyester/BW core–double sheath fibers containing one of these 8-hydroxyquinoline derivatives were tested in contact with the following model phytopathogenic microorganisms: *Pseudomonas corrugata* (*P. corrugata*), *Fusarium graminearum* (*F. graminearum*), and *Fusarium avenaceum* (*F. avenaceum*) [108]. As seen from Figure 14, the NQ-containing mats exhibited well-defined zones of inhibition upon contact with the pathogenic microorganisms. A comparable outcome was attained for fibrous materials containing CQ. This provides evidence that NQ and CQ incorporated into core–sheath or core–double sheath fibers preserve their antimicrobial efficacy against phytopathogenic microorganisms and may offer prospective utility in green agriculture.

### 3.3. Other Applications

Wei et al. [56] have claimed that nanofibers prepared by single-nozzle spinneret electrospinning of homogeneous blend solutions based on a conductive polymer, such as PANI, have potential applications in electronic or optical nanowires and sensors. In the case of PANI/PC and PANI/PS core–sheath composite fibers, the formation of a core–sheath structure is expected to provide conductive pathways or charge carrier mobility along the fiber length due to the continuous PANI domains and to the induced orientation of PANI chains caused by the whipping motion during the electrospinning process. In this way, fibers with favorable conductivity can be obtained. In addition, PC and PS insulators can provide better mechanical properties to overcome the inherent brittleness of PANI. As a result, this type of core–sheath fiber combines the mechanical properties of the insulator and the conductivity of PANI.

The photophysical properties of core–sheath fibers from the polyfluorene derivative/PMMA pair with a core from polyfluorene derivative and a PMMA sheath have been evaluated [109]. The results obtained demonstrate that full color light-emitting fibers with high luminescence efficiency can be obtained from the polyfluorene derivative/PMMA pair. These core–sheath fibers have the potential to be utilized in the fabrication of sensors.

## 4. Conclusions

As evidenced by the provided summary, the preparation of core–sheath fibers via single-nozzle spinneret electrospinning of homogeneous blend solutions is still at an early stage of development. It is of interest due to the possibility of using conventional electrospinning equipment. This is a prerequisite for the single-nozzle spinneret electrospinning of homogeneous blend solutions to be readily applicable in industrial applications for the production of core–sheath fibers, for example, by needleless or multi-jet electrospinning. Another advantage is the absence of stabilizing agents that are harmful to humans and the environment. This makes the fibrous materials prepared by single-nozzle spinneret electrospinning of homogeneous blend solutions eco- and bio-friendly, and they can be used in a variety of applications, including biomedical practice, food packaging and preservation, and for the needs of green agriculture. With regard to the functionalization of the surface of fibers obtained by electrospinning, it is of significant interest to explore the potential for the formation of a sheath from non-electrospinnable polyelectrolytes of natural origin, such as chitosan, HA, and SA, as well as from polyelectrolytes of synthetic origin with intrinsic biological activity. As previously outlined, the single-nozzle spinneret electrospinning of homogeneous blend solutions represents a suitable and readily feasible approach to achieving this desired outcome. Future directions in the field of preparation of core–sheath fibers by single-nozzle spinneret electrospinning of homogeneous blend solutions should seek to gain deeper knowledge of the factors that govern the formation of fibers with this type of architecture. There is also interest in expanding research into the applications of the fibers obtained using this approach.

## Figures and Tables

**Table 1:** Polymer pairs, molar masses of the components, and the resulting fiber structure, as referenced in ref. [83].

Polymer Pair	Molar Mass of the One Polymer Component	Molar Mass of the Second Polymer Component	Fiber Structure
PB/PS = 25/75 (*w*/*w*)	420,000 g/mol (PB)	280,000 g/mol (PS)	co-continuous
PMMA/PS = 25/75 (*w*/*w*)	15,000 g/mol or 120,000 g/mol (PMMA)	280,000 g/mol (PS)	co-continuous
PB/PMMA = 25/75 (*w*/*w*)	420,000 g/mol (PB)	15,000 g/mol or 120,000 g/mol (PMMA)	co-continuous
PB/PC = 25/75 (*w*/*w*)	420,000 g/mol (PB)	21,000 g/mol or 30,600 g/mol (PC)	core–sheath
PANI/PC = 25/75 (*w*/*w*)	65,000 g/mol (PANI)	21,000 g/mol or 30,600 g/mol (PC)	core–sheath
PMMA/PC = 25/75 (*w*/*w*)	15,000 g/mol or 120,000 g/mol (PMMA)	21,000 g/mol or 30,600 g/mol (PC)	core–sheath

**Table 2:** Macromolecular parameters of the water-soluble, non-ionogenic polymer and the polyelectrolyte used for preparation of core–sheath fibers by single-nozzle spinneret electrospinning of their homogeneous blend aqueous solutions described in ref. [98,99,100]; composition of the core and the sheath.

Non-Ionogenic Polymer Component	Polyelectrolyte Component	Core Composition	Sheath Composition
PVA ^(1)^ (98,000 g/mol, degree of hydrolysis = 88%)	CS ^(1)^ (3000 g/mol, 88% deacetylated)	PVA	CS
PEO ^(2)^ (900,000 g/mol)	CS ^(2)^ (3000, 10,000, 50,000, or 120,000 g/mol)	PEO	CS
PVP ^(1)^ (1,300,000 g/mol)	HA ^(1)^ (8700 g/mol)	HA	PVP
PVA ^(1)^ (98,000 g/mol, degree of hydrolysis = 88%)	HA ^(1)^ (8700 g/mol)	HA	PVA
PEO ^(1,3)^ (900,000 g/mol)	HA ^(1,3)^ (8700 g/mol)	HA	PEO
PVA ^(1)^ (98,000 g/mol, degree of hydrolysis = 88%)	SA ^(1)^ (1.28 Pa/s)	SA	PVA

^(1)^ polymers used in [98]; ^(2)^ [99]; or ^(3)^ [100].

## Data Availability

The data presented in this study are available upon request from the corresponding authors.

## References

[1] Xue J., Wu T., Dai Y., Xia Y. (2019). Electrospinning and Electrospun Nanofibers: Methods, Materials, and Applications. Chem. Rev..

[2] Medeiros G.B., Lima F.D.A., de Almeida D.S., Guerra V.G., Aguiar M.L. (2022). Modification and Functionalization of Fibers Formed by Electrospinning: A Review. Membranes.

[3] Tahir M., Vicini S., Sionkowska A. (2023). Electrospun Materials Based on Polymer and Biopolymer Blends—A Review. Polymers.

[4] Keirouz A., Wang Z., Reddy V.S., Nagy Z.K., Vass P., Buzgo M., Ramakrishna S., Radacsi N. (2023). The History of Electrospinning: Past, Present, and Future Developments. Adv. Mater. Technol..

[5] Avossa J., Herwig G., Toncelli C., Itel F., Rossi R.M. (2022). Electrospinning Based on Benign Solvents: Current Definitions, Implications and Strategies. Green Chem..

[6] Chen H., Chen X., Chen H., Liu X., Li J., Luo J., He A., Han C.C., Liu Y., Xu S. (2020). Molecular interaction, chain conformation, and rheological modification during electrospinning of hyaluronic acid aqueous solution. Membranes.

[7] Huang S., Mansouri J., Le-Clech P., Leslie G., Tang C.Y., Fane A.G. (2022). A Comprehensive Review of Electrospray Technique for Membrane Development: Current Status, Challenges, and Opportunities. J. Membr. Sci..

[8] Maleki H., Azimi B., Ismaeilimoghadam S., Danti S. (2022). Poly (lactic acid)-Based Electrospun Fibrous Structures for Biomedical Applications. Appl. Sci..

[9] Yang J., Xu L. (2023). Electrospun Nanofiber Membranes with Various Structures for Wound Dressing. Materials.

[10] Subramanian S., Muthumanickkam A., Sundaramoorthy S., Kubera Sampath Kumar S., Chavhan M.V. (2024). Textile Materials and Structures for Health and Well-Being: An Overview. Textile Materials for Good Health and Wellbeing.

[11] Kumar S.K.S., Subramanian S., Kumar K.H., Sundaramoorthy S., Kubera Sampath Kumar S., Chavhan M.V. (2024). Textile-Based Wound Dressings. Textile Materials for Good Health and Wellbeing.

[12] Gürtler A.-L., Rades T., Heinz A. (2023). Electrospun Fibers for the Treatment of Skin Diseases. J. Control Release.

[13] Tamilarasi G.P., Sabarees G., Manikandan K., Gouthaman S., Alagarsamy V., Solomon V.R. (2023). Advances in Electrospun Chitosan Nanofiber Biomaterials for Biomedical Applications. Mater. Adv..

[14] Zhang X., Wang Y., Gao Z., Mao X., Cheng J., Huang L., Tang J. (2024). Advances in Wound Dressing Based on Electrospinning Nanofibers. J. Appl. Polym. Sci..

[15] Abdulhussain R., Adebisi A., Conway B.R., Asare-Addo K. (2023). Electrospun Nanofibers: Exploring Process Parameters, Polymer Selection, and Recent Applications in Pharmaceuticals and Drug Delivery. J. Drug Deliv. Sci. Technol..

[16] Sadeghi-Aghbash M., Rahimnejad M., Adeli H., Feizi F. (2023). Wound Healing: An Overview of Wound Dressings on Health Care. Curr. Pharm. Biotechnol..

[17] Huang T., Zeng Y., Li C., Zhou Z., Xu J., Wang L., Yu D.-G., Wang K. (2024). Application and Development of Electrospun Nanofiber Scaffolds for Bone Tissue Engineering. ACS Biomater. Sci. Eng..

[18] Kuang G., Lin X., Li J., Sun W., Zhang Q., Zhao Y. (2024). Electrospun Nanofibers-Derived Functional Scaffolds for Cancer Therapy. Chem. Eng. J..

[19] Nayl A.A., Abd-Elhamid A.I., Awwad N.S., Abdelgawad M.A., Wu J., Mo X., Gomha S.M., Aly A.A., Bräse S. (2022). Review of the Recent Advances in Electrospun Nanofibers Applications in Water Purification. Polymers.

[20] Rajabifar N., Rostami A., Afshar S., Mosallanezhad P., Zarrintaj P., Shahrousvand M., Nazockdast H. (2024). Wound Dressing with Electrospun Core-Shell Nanofibers: From Material Selection to Synthesis. Polymers.

[21] Kopańska A., Brzeziński M., Draczyński Z. (2024). Combination of Polylactide with Cellulose for Biomedical Applications: A Recent Overview. Cellulose.

[22] Colín-Orozco J., Colín-Orozco E., Valdivia-Barrientos R. (2024). Production of Nanofibers by Electrospinning as Carriers of Agrochemical. Fibers.

[23] Chen J., Rong F., Xie Y. (2023). Fabrication, Microstructures and Sensor Applications of Highly Ordered Electrospun Nanofibers: A Review. Materials.

[24] Zhang C., Li Y., Wang P., Zhang H. (2020). Electrospinning of nanofibers: Potentials and Perspectives for Active Food Packaging. Compr. Rev. Food Sci. Food Saf..

[25] Taokaew S., Chuenkaek T. (2024). Developments of Core/Shell Chitosan-Based Nanofibers by Electrospinning Techniques: A Review. Fibers.

[26] Wang T., Su E. (2024). Electrospinning Meets Food Packaging: A Promising Pathway Towards Novel Opportunities in Food Preservation. Food Packag. Shelf Life.

[27] Reddy V.S., Tian Y., Zhang C., Ye Z., Roy K., Chinnappan A., Ramakrishna S., Liu W., Ghosh R. (2021). A Review on Electrospun Nanofibers Based Advanced Applications: From Health Care to Energy Devices. Polymers.

[28] Abdelhakeem E., Monir S., Teaima M.H.M., Rashwan K.O., El-Nabarawi M. (2023). State-of-the-Art Review of Advanced Electrospun Nanofiber Composites for Enhanced Wound Healing. AAPS Pharm. Sci. Tech..

[29] Stramarkou M., Tzegiannakis I., Christoforidi E., Krokida M. (2024). Use of Electrospinning for Sustainable Production of Nanofibers: A Comparative Assessment of Smart Textiles-Related Applications. Polymers.

[30] Wang Y., Liu L., Zhu Y., Wang L., Yu D.-G., Liu L.-Y. (2023). Tri-Layer Core–Shell Fibers from Coaxial Electrospinning for a Modified Release of Metronidazole. Pharmaceutics.

[31] SalehHudin H.S., Mohamad E.N., Mahadi W.N.L., Muhammad Afifi A. (2018). Multiple-Jet Electrospinning Methods for Nanofiber Processing: A Review. Mater. Manuf. Process.

[32] Ahmadi Bonakdar M., Rodrigue D. (2024). Electrospinning: Processes, Structures, and Materials. Macromol.

[33] Ramesh V.H., Goudanavar P., Ramesh B., Naveen N.R., Gowthami B. (2024). Pharmaceutical/Biomedical Applications of Electrospun Nanofibers-Comprehensive Review, Attentive to Process Parameters and Patent Landscape. Pharm. Nanotechnol..

[34] Forgie J.R.P., Leclinche F., Dréan E., Dolez P.I. (2023). Electrospinning of High-Performance Nanofibres: State of the Art and Insights into the Path Forward. Appl. Sci..

[35] Mao Y., Shen W., Wu S., Ge X., Ao F., Ning Y., Luo Y., Liu Z. (2023). Electrospun Polymers: Using Devices to Enhance Their Potential for Biomedical Applications. React. Funct. Polym..

[36] Haider A., Haider S., Kang I.-K. (2018). A Comprehensive Review Summarizing the Effect of Electrospinning Parameters and Potential Applications of Nanofibers in Biomedical and Biotechnology. Arab. J. Chem..

[37] Partheniadis I., Nikolakakis I., Laidmäe I., Heinämäki J. (2020). A Mini-Review: Needleless Electrospinning of Nanofibers for Pharmaceutical and Biomedical Applications. Processes.

[38] Kouhi M., Mobasheri M., Valipouri A., Kargari A., Matsuura T., Shirazi M.A. (2023). Needleless Electrospinning. Electrospun and Nanofibrous Membranes.

[39] Kancheva M., Toncheva A., Manolova N., Rashkov I. (2014). Advanced Centrifugal Electrospinning Setup. Mater. Lett..

[40] Zhang Y., Wang P., Shi Q., Ning X., Zheng J., Long Y.-Z. (2024). Research Progress and Prospect of Centrifugal Electrospinning and its Application. J. Alloys Compd..

[41] Nachev N., Spasova M., Manolova N., Rashkov I., Naydenov M. (2022). Electrospun Polymer Materials with Fungicidal Activity: A Review. Molecules.

[42] Aghjeha M.K.R., Razavi M.J., Silberschmidt V.V. (2022). Effect of interfibre bonding on mechanical behaviour of electrospun fibrous mats. Mechanics of Fibrous Networks.

[43] Montoya Y., Cardenas J., Bustamante J., Valencia R. (2021). Effect of Sequential Electrospinning and Co-electrospinning on Morphological and Fluid Mechanical Wall Properties of Polycaprolactone and Bovine Gelatin Scaffolds, for Potential Use in Small Diameter Vascular Grafts. Biomater. Res..

[44] Li X., Xu T., Liang Z., Amar V.S., Huang R., Maddipudi B.K., Shende R.V., Fong H. (2021). Simultaneous Electrospinning and Electrospraying for the Preparation of a Precursor Membrane Containing Hydrothermally Generated Biochar Particles to Produce the Value-Added Product of Carbon Nanofibrous Felt. Polymers.

[45] Ignatova M., Manolova N., Rashkov I. (2013). Electrospun Antibacterial Chitosan-Based Fibers. Macromol. Biosci..

[46] Abdullah M.F., Nuge T., Andriyana A., Ang B.C., Muhamad F. (2019). Core–Shell Fibers: Design, Roles, and Controllable Release Strategies in Tissue Engineering and Drug Delivery. Polymers.

[47] Sperling L.E., Reis K.P., Pranke P., Wendorff J.H. (2016). Advantages and Challenges Offered by Biofunctional Core-Shell Fiber Systems for Tissue Engineering and Drug Delivery. Drug Discov. Today.

[48] Zhang M., Ahmed A., Xu L. (2023). Electrospun Nanofibers for Functional Food Packaging Application. Materials.

[49] Elahi M.F., Lu W., Guoping G., Khan F. (2013). Core-shell Fibers for Biomedical Applications-A Review. J. Bioengineer. Biomed. Sci..

[50] Yarin A.L. (2011). Coaxial Electrospinning and Emulsion Electrospinning of Core-Shell Fibers. Polym. Adv. Technol..

[51] Li C., Li Q., Ni X., Liu G., Cheng W., Han G. (2017). Coaxial Electrospinning and Characterization of Core-Shell Structured Cellulose Nanocrystal Reinforced PMMA/PAN Composite Fibers. Materials.

[52] Mahalingam S., Matharu R., Homer-Vanniasinkam S., Edirisinghe M. (2020). Current Methodologies and Approaches for the Formation of Core-Sheath Polymer Fibers for Biomedical Applications. Appl. Phys. Rev..

[53] Li D., Yue G., Li S., Liu J., Li H., Gao Y., Liu J., Hou L., Liu X., Cui Z. (2022). Fabrication and Applications of Multi-Fluidic Electrospinning Multi-Structure Hollow and Core–Shell Nanofibers. Engineering.

[54] Ma L., Shi X., Zhang X., Li L. (2019). Electrospinning of Polycaprolacton/Chitosan Core-Shell Nanofibers by a Stable Emulsion System. Colloids Surf. A Physicochem. Eng. Asp..

[55] Ghosh S., Yadav A., Gurave P.M., Srivastava R.K. (2023). Unique Fiber Morphologies from Emulsion Electrospinning—A Case Study of Poly(ε-caprolactone) and Its Applications. Colloids Interfaces.

[56] Wei M., Lee J., Kang B., Mead J. (2005). Preparation of Core-Sheath Nanofibers from Conducting Polymer Blends. Macromol. Rapid Commun..

[57] Won J.J., Nirmala R., Navamathavan R., Kim H.Y. (2012). Electrospun Core-Shell Nanofibers from Homogeneous Solution of Poly (vinyl alcohol)/Bovine serum albumin. Int. J. Biol. Macromol..

[58] Huang W., Wang M.-J., Liu C.-L., You J., Chen S.-C., Wang Y.-Z., Liu Y. (2014). Phase Separation in Electrospun Nanofibers Controlled by Crystallization Induced Self-assembly. J. Mater. Chem. A..

[59] Wang M., Fang D., Wang N., Jiang S., Nie J., Yu Q., Ma G. (2014). Preparation of PVDF/PVP Core-Shell Nanofibers Mats via Homogeneous Electrospinning. Polymer.

[60] Liu D., Shi Q., Jin S., Shao Y., Huang J. (2019). Self-assembled Core-Shell Structured Organic Nanofibers Fabricated by Single-Nozzle Electrospinning for Highly Sensitive Ammonia Sensors. InfoMat.

[61] Kyuchyuk S., Paneva D., Karashanova D., Markova N., Georgieva A., Toshkova R., Manolova N., Rashkov I. (2022). Core-Sheath-Like Poly (ethylene oxide)/Beeswax Composite Fibers Prepared by Single-Spinneret Electrospinning. Antibacterial, Antifungal, and Antitumor Activities. Macromol. Biosci..

[62] Kyuchyuk S., Paneva D., Manolova N., Rashkov I., Karashanova D., Markova N. (2022). Core/Double-Sheath Composite Fibers from Poly(ethylene oxide), Poly(L-lactide) and Beeswax by Single-Spinneret Electrospinning. Polymers.

[63] Kyuchyuk S., Paneva D., Manolova N., Rashkov I., Karashanova D., Markova N. (2024). Composite Core-Double Sheath Fibers Based on Some Biodegradable Polyesters Obtained by Self-Organization During Electrospinning. J. Appl. Polym. Sci..

[64] Jiang H., Wang L., Zhu K. (2014). Coaxial Electrospinning for Encapsulation and Controlled Release of Fragile Water-Soluble Bioactive Agents. J. Control Release.

[65] Han D., Steckl A.J. (2019). Coaxial Electrospinning Formation of Complex Polymer Fibers and Their Applications. ChemPlusChem.

[66] Rathore P., Schiffman J.D. (2021). Beyond the Single-Nozzle: Coaxial Electrospinning Enables Innovative Nanofiber Chemistries, Geometries, and Applications. ACS Appl. Mater. Interfaces.

[67] Han D., Steckl A.J. (2017). Selective pH-Responsive Core-sheath Nanofiber Membranes for Chem/bio/med applications: Targeted Delivery of Functional Molecules. ACS Appl. Mater. Interfaces.

[68] Duan G., Greiner A. (2019). Air-Blowing-Assisted Coaxial Electrospinning toward High Productivity of Core/Sheath and Hollow Fibers. Macromol. Mater. Eng..

[69] Naeimirad M., Zadhoush A., Kotek R., Esmaeely Neisiany R., Nouri Khorasani S., Ramakrishna S. (2018). Recent advances in core/shell bicomponent fibers and nanofibers: A review. J. Appl. Polym. Sci..

[70] Matveev D.N., Anokhina T.S., Volkov V.V., Borisov I.L., Volkov A.V. (2023). Fabrication of Hollow Fiber Membranes: Effect of Process Parameters (Review). Membr. Membr. Technol..

[71] Yu D.-G., Li X.-Y., Wang X., Yang J.-H., Bligh S.W.A., Williams G.R. (2015). Nanofibers Fabricated Using Triaxial Electrospinning as Zero Order Drug Delivery Systems. ACS Appl. Mater. Interfaces.

[72] Chang S., Wang M., Zhang F., Liu Y., Liu X., Yu D.-G., Shen H. (2020). Sheath-Separate-Core Nanocomposites Fabricated Using a Trifluid Electrospinning. Mater. Des..

[73] Zhang C., Feng F., Zhang H. (2018). Emulsion Electrospinning: Fundamentals, Food Applications and Prospects. Trends Food Sci. Technol..

[74] Mouro C., Gomes A.P., Gouveia I.C. (2023). Emulsion Electrospinning of PLLA/PVA/Chitosan with *Hypericum perforatum* L. as an Antibacterial Nanofibrous Wound Dressing. Gels.

[75] Cheng H., Yang X., Che X., Yang M., Zhai G. (2018). Biomedical Application and Controlled Drug Release of Electrospun Fibrous Materials. Mater. Sci. Eng. C.

[76] Nikmaram N., Roohinejad S., Hashemi S., Koubaa M., Barba F.J., Abbaspourrad A., Greiner R. (2017). Emulsion-Based Systems for Fabrication of Electrospun Nanofibers: Food, Pharmaceutical and Biomedical Applications. RSC Adv..

[77] Norouzi M., Shabani I., Ahvaz H.H., Soleimani M. (2015). PLGA/Gelatin Hybrid Nanofibrous Scaffolds Encapsulating EGF for Skin Regeneration. J. Biomed. Mater. Res. Part A.

[78] Pal J., Skrifvars M., Nandan B., Srivastava R.K. (2017). Electrospun Composite Matrices from Tenside-Free Poly(ε-caprolactone)-Grafted Acrylic acid/Hydroxyapatite Oil-in-Water Emulsions. J. Mater. Sci..

[79] Nageeb El-Helaly S., Abd-Elrasheed E., Salim S.A., Fahmy R.H., Salah S., EL-Ashmoony M.M. (2021). Green Nanotechnology in the Formulation of a Novel Solid Dispersed Multilayered Core-Sheath Raloxifene-Loaded Nanofibrous Buccal Film; In Vitro and In Vivo Characterization. Pharmaceutics.

[80] Pal J., Sharma S., Sanwaria S., Kulshreshtha R., Nandan B., Srivastava R.K. (2014). Conducive 3D Porous Mesh of Poly(ε-caprolactone) Made via Emulsion Electrospinning. Polymer.

[81] Gouveia I.C., Mouro C., Essa K.S., Mahmoud K.H. (2024). Development of Drug-Delivery Textiles Using Different Electrospinning Techniques: A Review. Electrospinning-Theory, Applications, and Update Challenges.

[82] Wei M., Kang B., Sung C., Mead J., Reneker D.H., Fong H. (2006). Preparation of Nanofibers with Controlled Phase Morphology from Electrospinning of Polybutadiene-Polycarbonate Blends. Polymeric Nanofibers.

[83] Wei M., Kang B., Sung C., Mead J. (2006). Core-Sheath Structure in Electrospun Nanofibers from Polymer Blends. Macromol. Mater. Eng..

[84] Jiang Y., Fang D., Song G., Nie J., Chen B., Ma G. (2013). Fabrication of Core-Shell Nanofibers by Single Capillary Electrospinning Combined with Vapor Induced Phase Separation. New J. Chem..

[85] Niu Q., Zeng L., Mu X., Nie J., Ma G. (2016). Preparation and Characterization of Core-Shell Nanofibers by Electrospinning Combined with In Situ UV Photopolymerization. J. Ind. Eng. Chem..

[86] Niu Q., Mu X., Nie J., Ma G. (2016). Potential Fabrication of Core-Shell Electrospun Nanofibers from a Two-Step Method: Electrospinning and Photopolymerization. J. Ind. Eng. Chem..

[87] Ruguo Z., Hua Z., Hong Z., Ying F., Kun L., Wenwen Z. (2011). Thermal Analysis of Four Insect Waxes Based on Differential Scanning Calorimetry (DSC). Procedia Eng..

[88] Janesch J., Arminger B., Gindl-Altmutter W., Hansmann C. (2020). Superhydrophobic Coatings on Wood Made of Plant Oil and Natural Wax. Prog. Org. Coat..

[89] Delgado M.Z., Aranda F.L., Hernandez-Tenorio F., Garrido-Miranda K.A., Meléndrez M.F., Palacio D.A. (2024). Polyelectrolytes for Environmental, Agricultural, and Medical Applications. Polymers.

[90] Shikhi-Abadi P.G., Irani M. (2021). A Review on the Applications of Electrospun Chitosan Nanofibers for the Cancer Treatment. Int. J. Biol. Macromol..

[91] Helsper S., Singlar N., Garcia A.G., Liberatore M.W. (2024). Viscosity Scaling and Entangled Solution Rheology in Aqueous and Salt Solutions of Polyelectrolytes Containing Diallyl Dimethylammonium Groups. Rheol. Acta.

[92] Beleño Acosta B., Advincula R.C., Grande-Tovar C.D. (2023). Chitosan-Based Scaffolds for the Treatment of Myocardial Infarction: A Systematic Review. Molecules.

[93] Kumar M., Hilles A.R., Ge Y., Bhatia A., Mahmood S. (2023). A Review on Polysaccharides Mediated Electrospun Nanofibers for Diabetic Wound Healing: Their Current Status with Regulatory Perspective. Int. J. Biol. Macromol..

[94] Qasim S.B., Zafar M.S., Najeeb S., Khurshid Z., Shah A.H., Husain S., Rehman I.U. (2018). Electrospinning of Chitosan-Based Solutions for Tissue Engineering and Regenerative Medicine. Int. J. Mol. Sci..

[95] Li Y., Chen F., Nie J., Yang D. (2012). Electrospun Poly (Lactic Acid)/Chitosan Core-Shell Structure Nanofibers from Homogeneous Solution. Carbohydr. Polym..

[96] Cao L., Qu C., Liu J., Li W., Jiang L., Jing B., Wu C., Liu J. (2024). Functional Molecules Surface Segregation Engineering in Electrospinning: Design, regulation, and applications. Chem. Eng. J..

[97] Zhang J.-F., Yang D.-Z., Xu F., Zhang Z.-P., Yin R.-X., Nie J. (2009). Electrospun Core-Shell Structure Nanofibers from Homogeneous Solution of Poly (ethylene oxide)/Chitosan. Macromolecules.

[98] Mu X., Liu Y., Fang D., Wang Z., Nie J., Ma G. (2012). Electric Field Induced Phase Separation on Electrospinning Polyelectrolyte Based Core-Shell Nanofibers. Carbohydr. Polym..

[99] Chen G., Fang D., Wang K., Nie J., Ma G. (2015). Core-Shell Structure PEO/CS Nanofibers Based on Electric Field Induced Phase Separation via Electrospinning and Its Application. J. Polym. Sci. Part A Polym. Chem..

[100] Chen G., Guo J., Nie J., Ma G. (2016). Preparation, Characterization, and Application of PEO/HA Core Shell Nanofibers Based on Electric Field Induced Phase Separation during Electrospinning. Polymer.

[101] Yildiz A., Kara A.A., Acartürk F. (2020). Peptide-Protein Based Nanofibers in Pharmaceutical and Biomedical Applications. Int. J. Biol. Macromol..

[102] Xu T., Yang H., Yang D., Yu Z.-Z. (2017). Polylactic Acid Nanofiber Scaffold Decorated with Chitosan Islandlike Topography for Bone Tissue Engineering. ACS Appl. Mater. Interfaces.

[103] Cherdtrakulkiat R., Boonpangrak S., Sinthupoom N., Prachayasittikul S., Ruchirawat S., Prachayasittikul V. (2016). Derivatives (Halogen, Nitro and Amino) of 8-Hydroxyquinoline with Highly Potent Antimicrobial and Antioxidant Activities. Biochem. Biophys. Reports.

[104] Abouelhassan Y., Yang Q., Yousaf H., Nguyen M.T., Rolfe M., Schultz G.S., Huigens III R.W. (2017). Nitroxoline: A Broad-Spectrum Biofilm-Eradicating Agent against Pathogenic Bacteria. Int. J. Antimicrob. Agents.

[105] Pippi B., Reginatto P., Machado G.R.M., Bergamo V.Z., Lana D.F.D., Teixeira M.L., Franco L.L., Alves R.J., Andrade S.F., Fuentefria A.M. (2017). Evaluation of 8-Hydroxyquinoline Derivatives as Hits for Antifungal Drug Design. Med. Mycol. J..

[106] Jiang H., Taggart J.E., Zhang X., Benbrook D.M., Lind S.E., Ding W.-Q. (2011). Nitroxoline (8-Hydroxy-5-nitroquinoline) is More a Potent Anti-cancer Agent than Clioquinol (5-Chloro-7-iodo-8-quinoline). Cancer Lett..

[107] Yin X.D., Sun Y., Lawoe R.K., Yang G.-Z., Liu Y.-Q., Shang X.-F., Liu H., Yang Y.-D., Zhu J.-K., Huang X.-L. (2019). Synthesis and Anti-Phytopathogenic Activity of 8-Hydroxyquinoline Derivatives. RSC Adv..

[108] Kyuchyuk S., Paneva D., Manolova N., Rashkov I., Karashanova D., Naydenov M., Markova N. (2023). Electrospun Fibers of Biocompatible and Biodegradable Polyesters, Poly (ethylene oxide) and Beeswax with Anti-Bacterial and Anti-Fungal Activities. Materials.

[109] Kuo C.-C., Lin C.-H., Chen W.-C. (2007). Morphology and Photophysical Properties of Light-Emitting Electrospun Nanofibers Prepared from Poly(fluorene) Derivative/PMMA Blends. Macromolecules.

